# The metabolic effects of post-sepsis feeding of meals with only essential amino acids

**DOI:** 10.1042/CS20250414

**Published:** 2026-06-01

**Authors:** Nicolaas E.P. Deutz, Gabriella A.M. Ten Have, Sarah A. Rice, Marina B.W. Horner, Ilaria Minussi, Marielle P.K.J. Engelen

**Affiliations:** 1 Center for Translational Research in Aging and Longevity, Depts of Kinesiology & Sport Management, Texas A&M University, College Station, U.S.A.; 2 Primary Care & Rural Medicine, Texas A&M University, College Station, U.S.A.

**Keywords:** amino acids, metabolism, nutrition, sepsis

## Abstract

Nutritional support is a vital component of the rehabilitation process following sepsis. Essential amino acids (EAA) are recognized as a critical component for stimulating anabolism. However, it remains unclear whether EAA supplementation alone is more effective than providing a complete profile of total amino acids (TAA). We studied amino acid (AA) metabolism in 51 catheterized pigs (±27 kg) using the pulse tracer approach over a 7-day recovery period following sepsis. Animals were randomly and blindly assigned to receive post-sepsis nutrition containing either an EAA or a TAA mixture based on the composition of pig muscle. Statistical analyses were performed using a generalized linear mixed model. Plasma concentrations of EAA increased substantially in the EAA group post-sepsis (interaction effect, *P*<0.001), while those of non-essential AA increased in both groups (time effect, *P*<0.001). Whole-body intracellular production of most AA was reduced on day 3 and remained low on day 7 post-sepsis, with only small differences observed between the two groups. On day 7 post-sepsis, pigs receiving only EAA showed significantly (all *P*<0.001) lower intracellular production of hydroxyproline (−36%), ornithine (−11%), and tryptophan (−20%) and higher production of arginine (66%), citrulline (18%), glutamine (18%), taurine (22%), and some EAA. The EAA group showed a significantly higher net protein breakdown post-sepsis. In this sepsis-recovery pig model, dietary supplementation of both EAA and TAA failed to normalize the intracellular production of AA. More importantly, providing only EAA resulted in a significantly greater net protein breakdown compared with the TAA group.

## Introduction

Sepsis remains a global health concern, with focus increasingly shifting from acute survival to the protracted recovery phase [1,2]. During this period, persistent muscle wasting and metabolic dysfunction are the primary drivers of loss of physical function, also referred to as ICU-acquired weakness [3]. This rapid loss of lean body mass is often exacerbated by sepsis-induced anorexia that further worsens clinical outcomes [4]. Since nutritional needs shift as catabolism persists, providing targeted nutritional care is essential to optimize recovery and prevent long-term quality of life impairment [5,6].

We have previously reported that sepsis infection significantly affects amino acid (AA) metabolism in both pigs [7–10] and humans [11–15]. A major contributing factor is the combined increase in protein breakdown and reduced protein intake, which is likely a response to inflammation and related changes in the immune system, including altered (either increased or reduced) cytokine levels [7,16]. During sepsis, we observed an enhanced intracellular release of AA, which subsequently increases their plasma concentrations [7]. This release also promotes downstream metabolism, such as the production of glutamine and glutamate. These metabolic alterations ultimately could impair the function of muscle and internal organs.

While changes in AA metabolism are well-documented, we recently [7] demonstrated that these intracellular metabolic changes are already present during the early phase of sepsis when changes in plasma AA concentrations remain small. However, it is currently unclear how quickly these metabolic changes resolve once the sepsis is successfully treated. Addressing this knowledge gap is essential to determine the optimal timing for nutritional interventions in patients rehabilitating from sepsis.

We previously developed a sepsis model in pigs by intravenous infusion of live bacteria *Pseudomonas aeruginosa* [17]. During sepsis, these pigs develop the physiological responses also observed in humans [15], such as increases in body temperature, heart rate, lactate [18], and changes in protein and arginine metabolism [8]. A reversible sepsis model was established by discontinuing the bacterial infusion 9 h after onset and administering antibiotics to achieve full recovery [19,20].

Post-sepsis rehabilitation should start once the infection is controlled and the inflammation subsides. Even though nutritional support is an important component of sepsis rehabilitation, providing immediate nutrition is challenging. Current clinical guidelines suggest that nutrition should be administered in increasing amounts [3]. This caution is necessary because the gastrointestinal tract and systemic metabolism must readapt after the period of reduced food intake and gut-barrier dysfunction typical of sepsis [3].

Essential amino acids (EAA) are considered the most essential component of nutrition for stimulating muscle anabolism [21]. Since EAA must be obtained from the diet, while non-essential amino acids (NEAA) can be synthesized endogenously, NEAA are not strictly required from the diet [22,23]. However, we have shown that during sepsis, the NEAA arginine becomes conditionally essential due to a reduced conversion of citrulline to arginine (*de novo* arginine production) [24,25]. Other researchers have also indicated that glutamine requirements may increase during sepsis [26]. The increased need for certain NEAA may stem from an impaired ability to convert EAA to NEAA post-sepsis [27], though this remains underexplored.

To measure intracellular AA kinetics during sepsis recovery in pigs, we introduced the pulse tracer approach [7,28]. The main advantage of this method is its ability to calculate the appearance rates of individual metabolites in both extracellular and intracellular compartments [29–31].

The objective of the present study was to determine the rate of intracellular metabolic recovery over a 7-day post-sepsis period and to evaluate the efficacy of providing either EAA or a complete mixture of total amino acids (TAA) on recovery. Therefore, we investigated recovery in a reversible sepsis pig model, in which nutritional support was provided incrementally (from 25% to 100% of regular intake). We hypothesized that a nutritional mixture containing NEAA would be more effective in stimulating protein synthesis than a mixture of EAA.

## Methods

### Animals

Female Yorkshire cross/domestic pigs (*n* = 51, weight: 20–25 kg) were used in our experimental studies. Pigs were housed in steel pens (2 × 3 m) within a controlled housing facility (large animal cubicle, room temperature 22°C–24°C, 12 h light–dark cycle). They received 1 kg of a standard diet (Harlan Teklad Vegetarian Pig/Sow Grower) per day and had water available *ad libitum*. The studies were performed at the Veterinary Medical Park of Texas A&M University (College Station, TX, U.S.A.).

### Surgery

Animals underwent a surgical procedure for catheter placement, as described in detail previously [8,17,32]. Briefly, a midline laparotomy was performed to place seven catheters for blood sampling into the abdominal aorta (2), portal vein, hepatic vein, splenic vein, and caval vein (2). A gastric tube was also placed 20 cm into the stomach via a purse-string suture. All catheters were tunneled to exit the abdominal wall and fixed in a harness to be safely manipulated. The study utilized blood samples obtained from the arterial catheters, and isotopes were infused via the caval vein catheters. The gastric tube was used for the nutritional interventions.

Preoperative and postoperative care were standardized. During the recovery period (7–10 days), animals were accustomed to a small, movable cage (0.9 × 0.5 × 0.3 m), where the experiments would later be performed in conscious animals. Food was provided twice daily.

### Experimental design

The experiment started after the surgical recovery period. On the day of sepsis induction, the experiment began at 8 am, 16 h after the pigs’ last food intake. At *t*  = 0 h, sepsis was induced by continuous infusion of *P. aeruginosa* (PA, 10^9^ CFU/ml/h) [8]. PA is a human strain (IRS 12-4-4) from Shriners Burns Institute, University of Texas Medical Branch, Galveston. Fluid resuscitation was provided for post-sepsis vasodilation and extravascular system fluid loss (Ringer’s lactate solution, 30 ml/kg bw/h) from *t* = 0 h [1,33]. Hemodynamics were continuously monitored to ensure the sepsis hyperdynamic state was reached. Our previous studies demonstrated an increase in heart rate, respiratory rate, and body temperature [18] and these changes in vitals as follows: body temperature increase of 2°C–3°C, respiratory rate increase, MPAP increase but <35 mmHg, and heart rate increase but <200 bpm [7, 17, 18]. Arterial glucose levels were measured every 15 min using Accu-Chek sticks (Aviva Plus, Roche) to ensure that plasma glucose levels remained within the target range of 80–120 mg/dl. If glucose levels dropped below this range, 50% dextrose was infused IV to reach 100 mg/dl. Nine hours after sepsis induction, the bacterial infusion was stopped, and the pigs were administered gentamicin (5 mg/kg body weight IV) diluted in 20 ml of saline. Once sufficiently recovered, they were transported back to the holding room for the subsequent 7 days of experimentation.

On day 1 of the sepsis recovery phase, nutritional intervention began by twice-daily bolus intragastric administration of one of the following two meals: EAA or a balanced mixture of free essential and non-essential amino acids (TAA). This was provided according to a randomized, blinded design (Supplementary Table S1). The AA profile for the complete intervention meals was based on the composition of pig muscle [34]. The amount of the meals was incrementally increased post-sepsis to simulate the limited food intake often observed in septic patients [35]. Metabolic testing was performed on days 3 and 7 of the study. At the conclusion of day 7, pigs were killed with 125 mg/kg pentobarbital sodium and 16 mg/kg phenytoin sodium (Euthanasol®) administered via the central vein catheter.

### Infusion and sampling protocol

Metabolic tests were performed at three time points: 4 days before sepsis (pre-sepsis), day 3, and day 7 post-sepsis. Before food was provided, the pigs were given a pulse IV infusion of approximately 25 ml of a fluid containing multiple stable isotopes of AA (Table 1). Blood was sampled immediately before the isotope pulse was administered and 10, 20, 30, 45, 60, and 120 min following the isotope pulse (Supplementary Figure S1).

**Table 1:** Composition of the infused stable tracers

	Amount of tracer (mg)	
L-Arginine [guanidino-^15^N_2_]	16.7	
L-Citrulline [5-^13^C;4,4,5,5-^2^H_2_]	6.5	
L-Glutamate [1,2,-^13^C_2_]	23.2	
L-Glutamine [^15^N_2_]	35.3	
Glycine [1-^13^C]	21.0	
L-Histidine [^15^N_3_]	3.6	
L-Hydroxyproline [2,5,5-^2^H_3_]	0.7	
L-Isoleucine [1-^13^C]	5.8	
L-Leucine [^13^C_6_]	6.7	
L-Methionine [1-^13^C]	3.4	
L-Methionine [methyl-D_3_]	3.5	
L-Ornithine [^13^C_5_]	5.6	
L-Phenylalanine [ring-^13^C_6_]	15.6	
tau-Methyl-L-Histidine [methyl-^2^H_3_]	3.0	
L-Taurine [1,2,-^13^C_2_]	3.3	
L-Tryptophan [indole-^2^H_5_]	4.2	
L-Tyrosine [ring-D_4_]	8.5	
L-Valine [^13^C_5_]	4.1	

Volume is about 25 ml, made isotonic with NaCl.

Collected blood samples were immediately chilled on ice and processed within 1 h, as previously described [8]. For AA concentration and enrichment analysis, heparinized blood was centrifuged at 4°C and 8000×***g*** for 5 min. A total of 250 μl of plasma aliquots was deproteinized by adding 25 μl of trichloroacetic acid solution (TCA; 33.3% w/w), snap frozen in liquid nitrogen, and stored at −80°C.

### Amino acid concentrations and enrichments

AA concentrations and stable isotopic enrichments (tracer-to-tracee ratios, TTR) were determined on AA fluorenylmethoxycarbonyl (Fmoc) derivatives by microanalytical HPLC (microLC 200, Eksigent, part of SCIEX, Foster City, CA, U.S.A.), followed by heated electrospray triple-quadrupole mass spectrometry (5500 QTRAP, SCIEX). Twenty microliters of the plasma supernatant after TCA deproteinization of plasma samples were combined with 20 μl of an internal standard solution and stored at −80°C. The internal standard contained a high-mass stable isotope of every AA for concentration measurements and a single heavy AA as a process control for enrichment measurements. Standardized calibrators were prepared in 0.1 M HCl. Those calibrating concentrations contained mixed commercial AA at known, increasing concentrations spanning the AA physiological range. Enrichment calibrators contained AA at constant, near-physiological concentrations to which AA heavy isotopic tracers were added with increasing known TTRs to cover the expected TTR range.

Prior to LC-MS, both internally standardized plasma samples and calibrator samples were thawed and reacted with 9-fluorenylmethoxycarbonyl (Fmoc) chloride, followed by neutralization. Each reaction liquor (a 10-fold plasma dilution) was injected (160 nl) onto a 0.5 × 100 mm i.d. micro LC column maintained at 50°C. The column was packed by Eksigent (part of SCIEX, Foster City, CA, U.S.A.) with HALO C18 2.7 μm, 90Å core-shell material (Advanced Molecular Technologies). Fmoc-AAs eluted over 5 min with a mobile phase flow rate of 24 μl/min. The percentage of acetonitrile increased from 35% to 85%, displacing a water solution containing 5% isopropanol (aq) and 15 mM ammonium acetate.

Detection was by heated electrospray triple quadrupole mass spectrometry, during which Fmoc is cleaved from AAs either within the (quadrupole 2) collision cell or, if further breakage of the AA is needed to minimize naturally occurring heavy-AA background or to distinguish otherwise nominally isobaric heavy AA, then instead within the electrospray source/Q0 lens region to regenerate free AA for subsequent collision-cell fragmentation. A single injection was used to analyze both the normal AA and its heavy tracer (or, for concentrations, the internal standard); thus, the ratio could be readily calculated between their digitally integrated mass-chromatographic peak areas. These are inputs for calculations described below.

### Calculations

According to the double isotope dilution experimental design, peak area ratios measured in sets of calibrators were plotted against the known enrichments or concentrations of the calibrators. Regression lines were fitted, and resulting linear models could then fully characterize actual TTRs or concentrations in a repeatedly injected *reference standard*, a pool of TCA-plasma supernatants from several blood samples. The reference standard was then included in all subsequent batches to carry forward the calibration [15]. For enrichments, the TTR was corrected for the background TTR, which was measured in plasma before administering the tracer pulse.

Individual TTR decay of every time point and study day was fitted with a 2-exponential TTR decay curve in GraphPad Prism with the formula (TTR = *a* × EXP(−*k*_1_ × *t*) + *b* × EXP(−*k*_2_ × *t*)). The fitted parameters with the robust fitting option, *a*, *b*, *k*_1_, and *k*_2_, were subsequently used to calculate the predicted whole-body production (WBP), which represents the rate of appearance of the AA into the extracellular pool, and the parameters of compartmental analysis [30,31] (Supplementary Table S2).

The compartmental analysis allows calculating the tracee fluxes between compartments using the information obtained from the tracer pulse [31]. We have identified two pools that we have hypothesized to be the extracellular (Q_1_) and intracellular (Q_2_) pool (Supplementary Figure S2). The amounts of tracee in the pools are Q_1_ and Q_2_. Flux to/from pool Q_2_ is either to pool 1 or out/in of pool Q_2_ (F_0,2_ = U_2_) from production or disposal into or out of the intracellular pool. Leading to calculated U_2_ (whole-body intracellular production). The amount in Q_1_ relates to differences in the plasma concentration when the Q_1_ pool size remains about the same. The unit of measure for Q_1_ and Q_2_ is μmol, and for WBP, F_0,2_, and U_2_ is μmol/min.

The conversion of an AA into another metabolite was calculated by using the WBP or F_0,2_ of the product AA, multiplied by the estimated ratio between the TTR of the product and the TTR of the substrate [29]. The conversion of citrulline to arginine is *de novo* arginine production. The conversion of phenylalanine to tyrosine is used as the proxy for net protein breakdown. ReMethylation is methionine produced *via* homocysteine degradation, calculated as the difference between the turnover, measured with l-methionine [methyl-D_3_] and l-methionine [1-^13^C]. The clearance of AA was calculated by the ratio between WBP and the AA plasma concentration expressed in liters/min.

High-sensitivity C-reactive protein (CRP) and cytokines were measured by the Milliplex analyzer, Luminex xMAP technology (LS400®), and cytokine assay. Plate PCYTMG-23K-13PX was used for the interleukins, granulocyte-macrophage colony-stimulating factor, and tumor-necrosis factor (TNF-a), and plate PAPP1-160K was used for haptoglobin and CRP (Millipore Sigma).

### Statistical analysis

Results are expressed as mean [95% confidence interval (95% CI)]. We did not remove any possible outliers or do any imputation for missing data. Statistical analysis was done by a generalized linear mixed model (Type III, maximal likelihood) with family: Gaussian and Link: Identify or Log with study group, study day, and pre-sepsis pig weight as fixed-effect variables and pig ID as a random-effect grouping factor. Post hoc testing was performed using Tukey correction. JASP (Version 0.19.3) [36] was used for statistical analysis, and GraphPad Prism (10.4) was used for the fitting of decay curves. The level of significance (α: 0.05) was tested two-sided.

## Results

### General characterization of pigs

**Figure 1:**
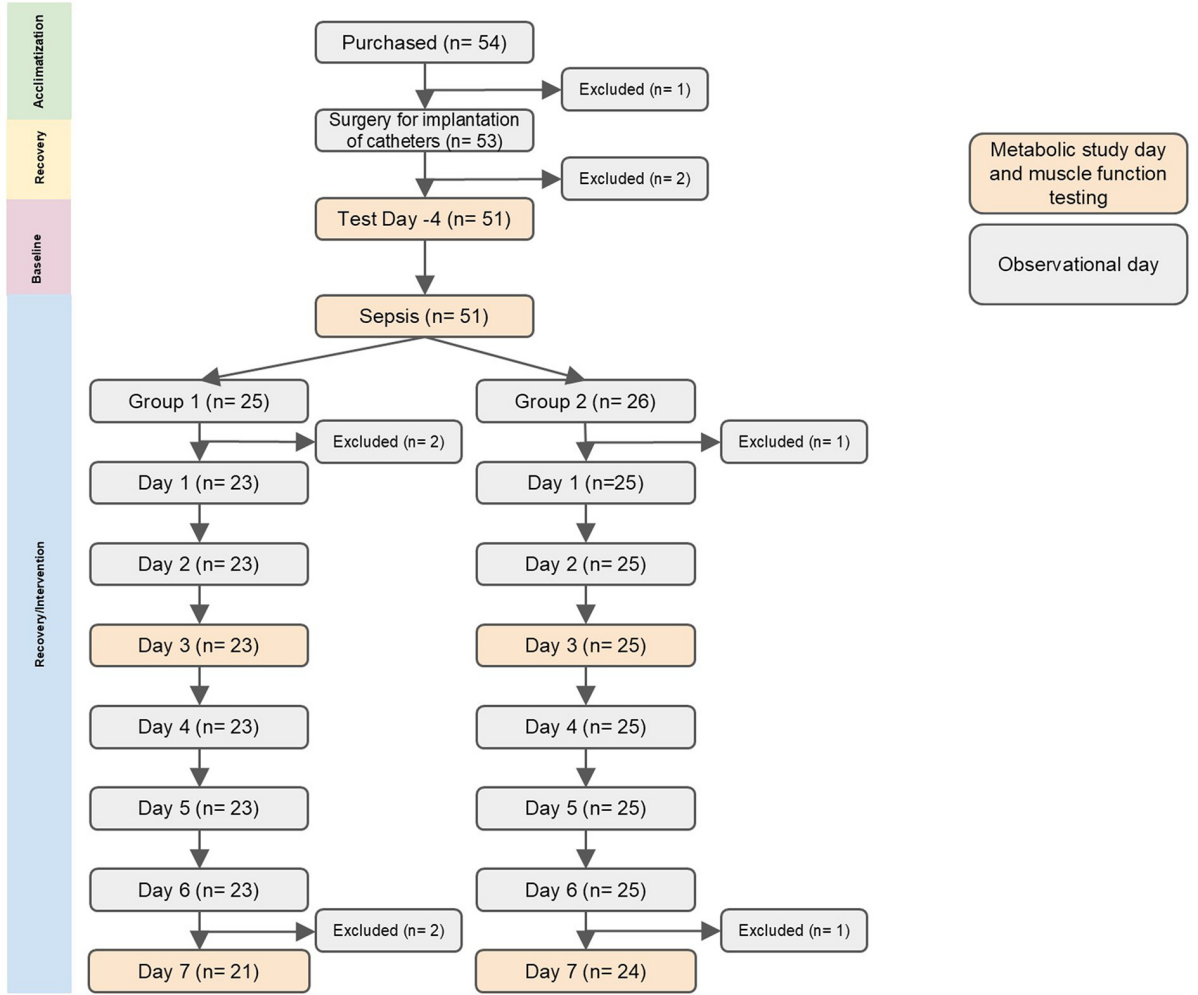
Consort Diagram Consort diagram demonstrating the number of animals per group and time point.

We studied 51 pigs with an average weight of approximately 27 kg. A total of six pigs died during the study: three pigs during the sepsis and three by day 7 post-sepsis (Figure 1). Pig weight (Table 2) was lower after sepsis, but by day 6 (Table 3), it was comparable with the pre-sepsis weight and did not differ between the EAA and TAA groups.

**Table 2:** Overview of the studied pigs

Phase	Pigs (*n*)		Weight (kg)		Body temperature (°C)	
	Group 1	Group 2	Group 1	Group 2	Group 1	Group 1
Pre-sepsis	25	26	27.6 (1.2)	27.3 (1.6)	38.7 (0.5)	38.8 (0.4)

Intervention	EAA	TAA	EAA	TAA	EAA	TAA
Day 3	23	25	26.3 (1.0)	26.6 (1.4)	38.1 (0.5)	38.3 (0.4)
Day 7	21	24	27.5 (1.3)	27.4 (1.9)	38.3 (0.2)	38.4 (0.4)

Data are mean (SD). Group 1 are the pigs that received EAA feeding and Group 2 are the pigs that received TAA feeding post-sepsis.

**Table 3:** Changes in weight and temperature of the studied pigs

Phase	Weight (kg)			Body Temperature (°C)		
	Group 1 *P* versus pre-sepsis	Group 2 *P* versus pre-sepsis	Group 1–Group 2	Group 1	Group 1	Group 1–Group 2
Intervention	EAA	TAA	EAA–TAA	EAA	TAA	EAA–TAA
Day 1	0.93 [0.30, 1.56] ***P* = 0.0057**	1.21 [0.57, 1.85] ***P* = 0.0007**	−0.01 [−0.98, 0.95] *P* = 0.9761	−0.04 [−0.60, 0.51] *P* = 0.8579	−0.09 [−0.47, 0.30] *P* = 0.6310	0.02 [−0.32, 0.36] *P* = 0.8971
Day 2	−1.04 [−1.39, −0.68] ***P*<0.0001**	−0.34 [−0.76, 0.07] *P* = 0.1018	−0.43 [−1.39, 0.53] *P =* 0.3819	−0.08 [−0.37, 0.21] *P* = 0.5753	−0.32 [−0.60, −0.03] ***P* = 0.0305**	0.17 [−0.10, 0.44] *P* = 0.2159
Day 3	−1.43 [−1.54, −0.94] ***P*<0.0001**	−0.66 [−1.07, −0.26] ***P* = 0.0025**	−0.32 [−1.28, 0.64] *P* = 0.5176	−0.61 [−1.23, 0.01] *P* = 0.0527	−0.48 [−0.84, −0.13] ***P* = 0.0126**	−0.12 [−0.45, 0.21] *P* = 0.4741
Day 4	−0.85 [−1.08, −0.63] ***P*<0.0001**	−0.71 [−0.99, −0.43] ***P*<0.0001**	0.12 [−0.84, 1.08] *P* = 0.7997	−0.13 [−0.42, 0.23] *P* = 0.3165	0.03 [−0.12, 0.18] *P* = 0.666	−0.06 [−0.21, 0.32] *P* >0.999
Day 5	−0.86 [−1.24, −0.48] ***P* = 0.0001**	−0.70 [−0.95, −0.45] ***P*<0.0001**	0.15 [−0.81, 1.1] *P* = 0.7572	−0.09 [−0.41, 0.23] *P* = 0.5719	−0.09 [−0.33, 0.14] *P* = 0.4110	0.05 [−0.21, 0.32] *P* = 0.6731
Day 6	−0.32 [−0.79, 0.15] *P* = 0.1666	−0.07 [−0.43, 0.28] *P* = 0.6785	0.01 [−0.96, 0.96] *P* = 0.9997	−0.23 [−0.65, 0.20] *P* = 0.2640	−0.03 [−0.40, 0.34] *P* = 0.8419	−0.19 [−0.46, 0.09] *P* = 0.1861
Day 7	−0.11 [−0.36, 0.13] *P* = 0.3490	0.15 [−0.25, 0.55] *P* = 0.4416	−0.04 [−1.01, 0.93] *P* = 0.9293	−0.41 [−1.01, 0.19] *P* = 0.1501	−0.35 [−0.71, −0.01] ***P* = 0.048**	−0.06 [−0.41, 0.28] *P* = 0.7176

Data are estimated differences in kg or Celsius from pre-sepsis values. Data are mean [95% CI] and estimated differences [95% CI]. Statistics (JASP) by linear mixed model. Post hoc testing *P*-values are corrected by Tukey. Bold is *P*<0.05. Group 1 are the pigs that received EAA feeding, and Group 2 are the pigs that received TAA feeding post-sepsis.

### Plasma amino acid concentrations

**Figure 2:**
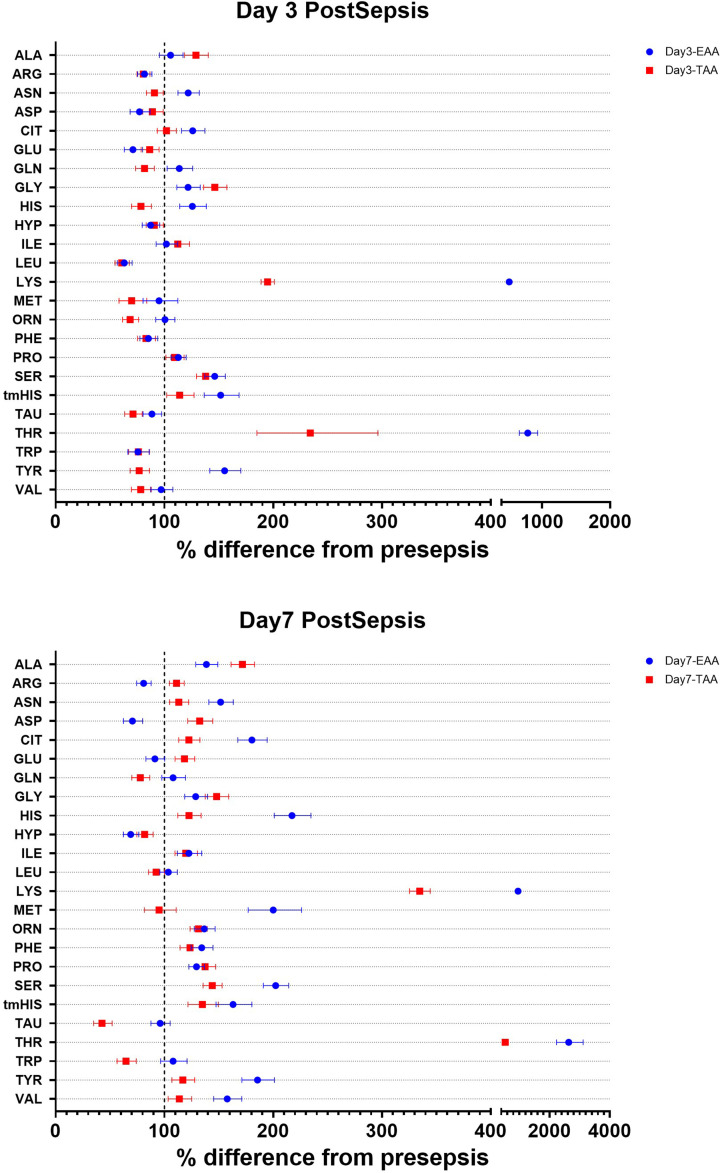
Changes in plasma concentrations Forest plots showing the changes in plasma amino acid concentrations at day 3 post-sepsis (top panel: A) and day 7 post-sepsis (bottom panel: B). Data are expressed as the percentage difference from presepsis and the 95% CI.

Plasma concentrations at day 3 post-sepsis (Figure 2 and Table 4) were lower compared with pre-sepsis in the TAA group for several amino acids, including arginine, glutamate, glutamine, histidine, leucine, methionine, ornithine, taurine, tryptophan, tyrosine, and valine. Conversely, concentrations were higher for alanine, glycine, lysine, serine, and threonine. The EAA group did not have higher plasma concentrations of most non-essential amino acids at day 3 post-sepsis, except for asparagine, citrulline, glutamine, ornithine, and tyrosine. The plasma concentrations of the essential amino acids histidine, lysine, and threonine were higher in the EAA compared with the TAA group. In addition, the EAA group showed a higher tau-methyl histidine and lower hydroxyproline plasma concentration.

**Table 4:** Plasma amino acid concentrations

Amino acid	*P* ANOVA	Pre-sepsis		Day 3 post-sepsis			Day 7 post-sepsis		
	Group time Group × Time	Group 1	Group 2	EAA	TAA	TAA–EAA	EAA	TAA	TAA–EAA
Alanine	<0.001 <0.001 0.004	322 [290, 358]	312 [281, 347]	340 [307, 377] *P* = 0.967	403 [370, 438] *P*<0.001	62.3 [16, 108.6] *P* = 0.017	447 [415, 480] *P≤*0.001	537 [504, 571] *P≤*0.001	89.8 [43.8, 135.9] *P≤*0.001
Arginine	<0.001 <0.001 <0.001	58 [54, 62]	63 [59, 67]	47 [44, 51] *P≤*0.001	51 [47, 55] *P*<0.001	3.7 [−1.5, 8.9] *P* = 0.294	47 [43, 51] *P≤*0.001	70 [66, 75] *P* = 0.024	23.6 [17.8, 29.5] *P≤*0.001
Asparagine	0.0027 <0.001 <0.001	66 [61, 72]	62 [57, 68]	81 [74, 87] *P≤*0.001	57 [52, 62] *P* = 0.0701	−24.1 [−32, −16.2] *P≤*0.001	100 [93, 108] *P≤*0.001	70 [65, 76] *P* = 0.0041	−29.9 [−39.2, −20.7] *P≤*0.001
Aspartate	0.0111 <0.001 <0.001	5.95 [5.39, 6.58]	5.63 [5.08, 6.23]	4.59 [4.07, 5.16] *P≤*0.001	5.01 [4.5, 5.57] *P* = 0.1996	0.42 [−0.33, 1.16] *P* = 0.4676	4.2 [3.71, 4.76] *P≤*0.001	7.46 [6.84, 8.13] *P≤*0.001	3.25 [2.42, 4.08] *P≤*0.001
Citrulline	0.005 <0.001 <0.001	50 [45, 55]	51 [47, 56]	63 [58, 68] *P≤*0.001	52 [48, 57] *P* = 0.998	−10.4 [−17.2, −3.7] *P* = 0.005	90 [83, 97] *P≤*0.001	63 [58, 68] *P≤*0.001	−27 [−35.3, −18.6] *P≤*0.001
Glutamate	0.012 <0.001 0.041	152 [141, 165]	143 [137, 149]	108 [96, 122] *P≤*0.001	124 [112, 136] *P* = 0.025	15.3 [−1.8, 32.4] *P* = 0.153	139 [127, 153] *P* = 0.577	169 [157, 183] *P* = 0.001	30.3 [11.8, 48.8] *P* = 0.003
Glutamine	1 0.904 <0.001	489 [441, 542]	481 [435, 533]	556 [501, 616] *P≤*0.001	393 [354, 437] *P≤*0.001	−162.4 [−237.6, −87.2] *P≤*0.001	528 [478, 584] *P* = 0.142	375 [338, 417] *P≤*0.001	−153.4 [−224.2, −82.5] *P≤*0.001
Glycine	0.002 <0.001 <0.001	637 [578, 702]	704 [645, 768]	775 [710, 847] *P≤*0.001	1030 [957, 1108] *P≤*0.001	254.6 [153.5, 355.8] *P≤*0.001	821 [757, 890] *P≤*0.001	1042 [969, 1121] *P≤*0.001	221.2 [119.9, 322.5] *P≤*0.001
Histidine	<0.001 <0.001 <0.001	49 [44, 54]	47 [43, 52]	61 [56, 67] *P≤*0.001	37 [33, 42] *P≤*0.001	−24.2 [−31.3, −17.1] *P≤*0.001	106 [98, 114] *P≤*0.001	58 [53, 63] *P≤*0.001	−48 [−57.6, −38.3] *P≤*0.001
Hydroxy proline	<0.001 <0.001 <0.001	67 [61, 73]	68 [62, 74]	58 [53, 64] *P* = 0.004	61 [56, 67] *P* = 0.074	3.2 [−4.1, 10.4] *P* = 0.626	46 [42, 51] *P≤*0.001	55 [51, 61] *P≤*0.001	9.3 [2.4, 16.2] *P* = 0.017
Isoleucine	<0.001 <0.001 0.018	76 [69, 84]	74 [67, 82]	78 [70, 86] *P* = 0.999	83 [76, 91] *P* = 0.06	5.6 [−4.1, 15.3] *P* = 0.45	94 [85, 102] *P≤*0.001	89 [81, 97] *P≤*0.001	−4.8 [−16.1, 6.4] *P* = 0.641
Leucine	0.152 <0.001 0.249	123 [114, 133]	121 [112, 131]	78 [70, 87] *P≤*0.001	73 [66, 82] *P≤*0.001	−4.4 [−15.4, 6.5] *P* = 0.672	128 [118, 138] *P* = 0.969	112 [103, 121] *P* = 0.456	−16.1 [−29.2, −3] *P* = 0.032
Lysine	<0.001 <0.001 <0.001	53 [51, 55]	54 [52, 56]	274 [265, 282] *P≤*0.001	105 [102, 108] *P≤*0.001	−168.7 [−174.5, −162.9] *P≤*0.001	508 [492, 525] *P≤*0.001	180 [175, 186] *P≤*0.001	−327.8 [−340.9, −314.6] *P≤*0.001
Methionine	0.0104 <0.001 <0.001	29 [25, 34]	32 [28, 38]	28 [23, 33] *P* = 0.9928	23 [19, 27] *P* = 0.0015	−5.1 [−10.5, 0.4] *P* = 0.1297	58 [52, 66] *P≤*0.001	31 [26, 36] *P* = 0.993	−27.5 [−35.4, −19.7] *P≤*0.001
Ornithine	0.0833 <0.001 <0.001	45 [41, 49]	53 [50, 57]	45 [41, 49] *P* = 1	36 [33, 41] *P≤*0.001	−8.7 [−14.1, −3.2] *P* = 0.0037	61 [57, 66] *P≤*0.001	70 [66, 74] *P≤*0.001	8.4 [2.4, 14.5] *P* = 0.0125
Phenylalanine	0.0332 <0.001 0.0098	50 [46, 55]	46 [42, 50]	43 [39, 47] *P* = 0.007	38 [34, 42] *P* = 0.005	−5 [−10.5, 0.4] *P* = 0.1338	67 [63, 73] *P≤*0.001	56 [52, 61] *P≤*0.001	−11.2 [−17.9, −4.5] *P* = 0.002
Proline	1 <0.001 0.123	161 [151, 172]	161 [149, 173]	182 [171, 194] *P* = 0.0013	176 [163, 190] *P* = 0.0554	−6.4 [−24.7, 12] *P* = 0.7463	209 [198, 221] *P≤*0.001	221 [206, 236] *P≤*0.001	11.8 [−8.5, 32] *P* = 0.4452
Serine	0.0066 <0.001 <0.001	93 [86, 100]	91 [85, 98]	136 [127, 145] *P≤*0.001	125 [118, 133] *P≤*0.001	−10.6 [−21.5, 0.2] *P* = 0.1071	188 [177, 199] *P≤*0.001	131 [123, 139] *P≤*0.001	−57 [−70.4, −43.6] *P≤*0.001
Tau-methyl- histidine	0.0102 <0.001 <0.001	6.28 [5.59, 7.06]	6.23 [5.54, 7]	9.53 [8.58, 10.59] *P≤*0.001	7.1 [6.36, 7.92] *P* = 0.0639	−2.44 [−3.64, −1.23] *P≤*0.001	10.25 [9.27, 11.34] *P≤*0.001	8.4 [7.58, 9.32] *P≤*0.001	−1.85 [−3.19, −0.51] *P* = 0.0137
Taurine	1 <0.001 1	94 [86, 103]	95 [86, 104]	83 [76, 92] *P* = 0.196	67 [60, 76] *P≤*0.001	−15.9 [−26.9, −4.9] *P* = 0.0095	90 [83, 99] *P* = 0.9869	40 [33, 49] *P≤*0.001	−50 [−61.6, −38.4] *P≤*0.001
Threonine	<0.001 <0.001 <0.001	81 [58, 113]	49 [32, 76]	636 [537, 753] *P* = 0.5441	114 [90, 145] *P≤*0.001	−521.3 [−632.4, −410.2] *P≤*0.001	2127 [1802, 2510] *P≤*0.001	261 [221, 309] *P≤*0.001	−1865.4 [−2220.5, −1510.2] *P≤*0.001
Tryptophan	0.037 <0.001 <0.001	22 [20, 25]	21 [19, 24]	17 [15, 19] *P≤*0.001	16 [14, 18] *P≤*0.001	−0.5 [−3.3, 2.3] *P* = 0.9243	24 [21, 27] *P* = 0.4897	14 [12, 16] *P≤*0.001	−10 [−13.3, −6.8] *P≤*0.001
Tyrosine	<0.001 <0.001 <0.001	31 [28, 35]	32 [29, 36]	49 [44, 53] *P≤*0.001	25 [22, 28] *P≤*0.001	−23.8 [−29.1, −18.5] *P≤*0.001	58 [54, 63] *P≤*0.001	38 [34, 41] *P* = 0.0248	−20.4 [−26.1, −14.6] *P≤*0.001
Valine	0.0011 <0.001 <0.001	170 [153, 189]	160 [145, 176]	165 [148, 183] *P* = 0.994	125 [111, 141] *P≤*0.001	−39.7 [−62.6, −16.7] *P* = 0.0014	268 [247, 291] *P≤*0.001	182 [165, 200] *P* = 0.1358	−86 [−114.9, −57.1] *P≤*0.001
BCAA	<0.001 <0.001 <0.001	379 [353, 407]	351 [325, 378]	325 [300, 352] *P* = 0.003	289 [266, 314] *P≤*0.001	−36.1 [−69.7, −2.5] *P* = 0.0695	493 [464, 525] *P≤*0.001	385 [359, 412] *P* = 0.2384	−108.9 [−148.6, −69.1] *P≤*0.001
EAA	<0.001 <0.001 <0.001	683 [622, 750]	622 [563, 687]	1415 [1323, 1513] *P≤*0.001	640 [580, 707] *P* = 0.9972	−775.3 [−864.3, −686.3] *P≤*0.001	3497 [3292, 3715] *P≤*0.001	1024 [947, 1108] *P≤*0.001	−2472.6 [−2670, −2275.2] *P≤*0.001
NEAA	1 <0.001 0.9749	1967 [1853, 2087]	2107 [1990, 2230]	2188 [2063, 2319] *P* = 0.0017	2442 [2317, 2574] *P≤*0.001	254.5 [70.3, 438.8] *P* = 0.0135	2460 [2334, 2592] *P≤*0.001	2703 [2571, 2841] *P≤*0.001	243.5 [55.1, 431.8] *P* = 0.0224
SumAA	<0.001 <0.001 <0.001	2801 [2627, 2986]	2812 [2623, 3016]	3802 [3592, 4024] *P≤*0.001	3102 [2910, 3307] *P* = 0.0084	−699.9 [−910.8, −489.1] *P≤*0.001	6135 [5818, 6469] *P≤*0.001	3756 [3531, 3995] *P≤*0.001	−2378.9 [−2703.7, −2054.2] *P≤*0.001
Tryptophan/ LNAA	0.8296 <0.001 <0.001	0.044 [0.038, 0.052]	0.044 [0.038, 0.052]	0.037 [0.031, 0.043] *P≤*0.001	0.042 [0.036, 0.049] *P* = 0.7234	0.005 [−0.003, 0.013] *P* = 0.4004	0.035 [0.029, 0.041] *P≤*0.001	0.026 [0.022, 0.031] *P≤*0.001	−0.009 [−0.016, −0.001] *P* = 0.0406
Tyrosine/ LNAA	0.1516 <0.001 <0.001	0.204 [0.183, 0.227]	0.21 [0.19, 0.232]	0.269 [0.244, 0.298] *P≤*0.001	0.202 [0.183, 0.222] *P* = 0.9509	−0.068 [−0.101, −0.035] *P≤*0.001	0.253 [0.23, 0.278] *P≤*0.001	0.225 [0.205, 0.247] *P* = 0.5494	−0.028 [−0.059, 0.004] *P* = 0.1672

Plasma concentrations are mean in μM [95% CI]. Statistics (JASP) by generalized linear mixed model (Family: Gaussian, Link: Log) with study group, study day, and pig weight as fixed effects variables and pig ID as random effect grouping factor. Post hoc testing *P*-values are corrected by Tukey. Group 1 are the pigs that received EAA feeding, and Group 2 are the pigs that received TAA feeding post-sepsis. *P* ANOVA is the *P*-value of the main effect group.

At day 7 post-sepsis, lower concentrations of glutamine, hydroxyproline, taurine, and tryptophan were still present compared with pre-sepsis in the TAA group. Compared with the TAA group, the EAA group had lower plasma concentrations of alanine, arginine, aspartate, glutamate, and hydroxyproline. In contrast, the EAA group showed higher plasma concentrations of asparagine, citrulline, glutamine, histidine, lysine, methionine, serine, tau methylhistidine, taurine, threonine, tryptophan, tyrosine, and valine. Many of the observed differences between the TAA and EAA cannot be easily attributed solely to the differences in meal composition of the meals.

We also calculated the ratio between the plasma concentrations of tryptophan or tyrosine and the large neutral amino acids (LNAA) (Table 4). These ratios are commonly used as an estimate of brain serotonin or dopamine levels [37]. A significant interaction between dietary treatment and time was observed for tryptophan/LNAA ratios. Reductions were noted in the EAA group on days 3 and 7 compared with pre-sepsis, whereas a reduction in the TAA group was only observed on day 7 post-sepsis. No changes were observed in the tyrosine/LNAA ratios throughout the study.

### Whole-body production of amino acids

**Figure 3:**
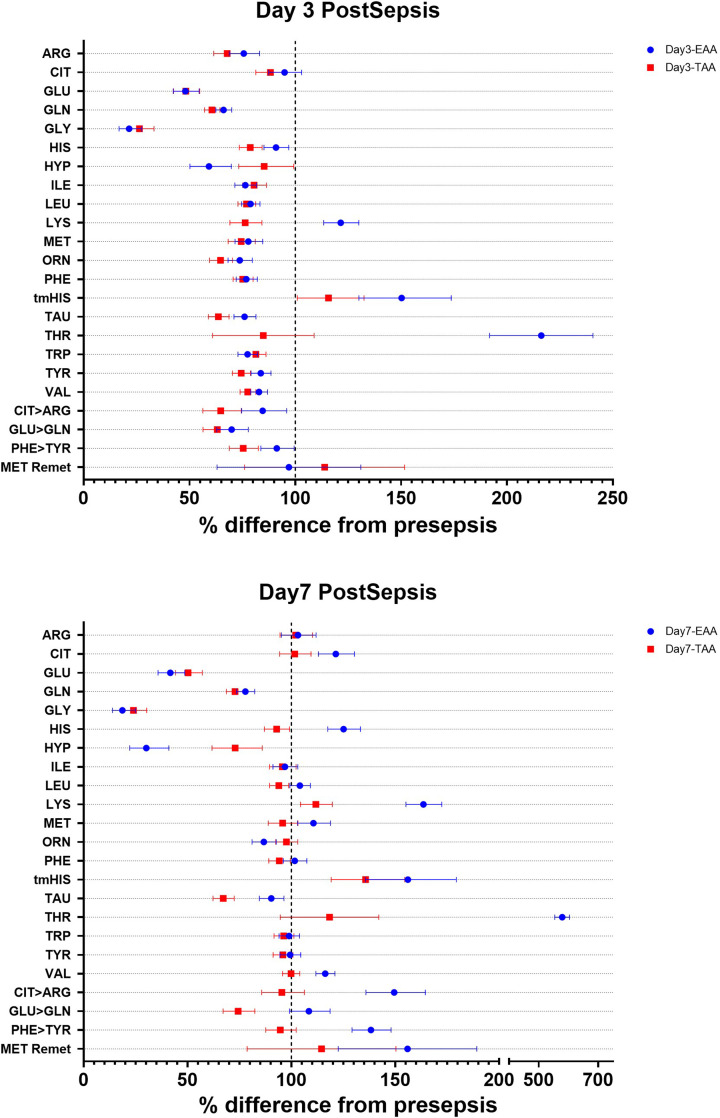
Changes in Whole Body Production (WBP) Forest plots showing the changes in WBP at day 3 post-sepsis (top panel: A) and day 7 post-sepsis (bottom panel: B). Data are expressed as the percentage difference from presepsis and the 95% CI.

In contrast with the changes observed in plasma amino acid concentrations, WBP of amino acids showed a distinct pattern. By day 3 post-sepsis, WBP for many of the amino acids had decreased (Figure 3 and Supplementary Table S3) in both EAA and TAA groups, except for tau-methyl-histidine in both groups and lysine in the TAA group, which were increased. A reduction in net protein breakdown was only observed in the TAA group. Comparisons between the EAA and TAA interventions revealed a few differences: The EAA group had higher WBP for arginine, glutamine, histidine, lysine, taurine, threonine, tyrosine, and valine compared with the TAA group. In addition, *de novo* arginine production (conversion of citrulline to arginine) and net protein breakdown (conversion of phenylalanine to tyrosine) were also higher.

By day 7, the WBP in the TAA group had normalized for several amino acids, though it remained substantially lower than pre-sepsis values for glutamate, glutamine, glycine, hydroxyproline, and taurine and higher for lysine and tau-methyl histidine. When comparing the groups at Day 7, the EAA group maintained a higher WBP of citrulline, histidine, leucine, lysine, methionine, phenylalanine, taurine, threonine, and valine but a lower WBP of hydroxyproline. On day 7, while *de novo* arginine production, glutamate to glutamine conversion, and net protein breakdown had normalized in the TAA group, they were substantially higher in the EAA group compared with pre-sepsis values.

### Clearance of amino acids

**Figure 4:**
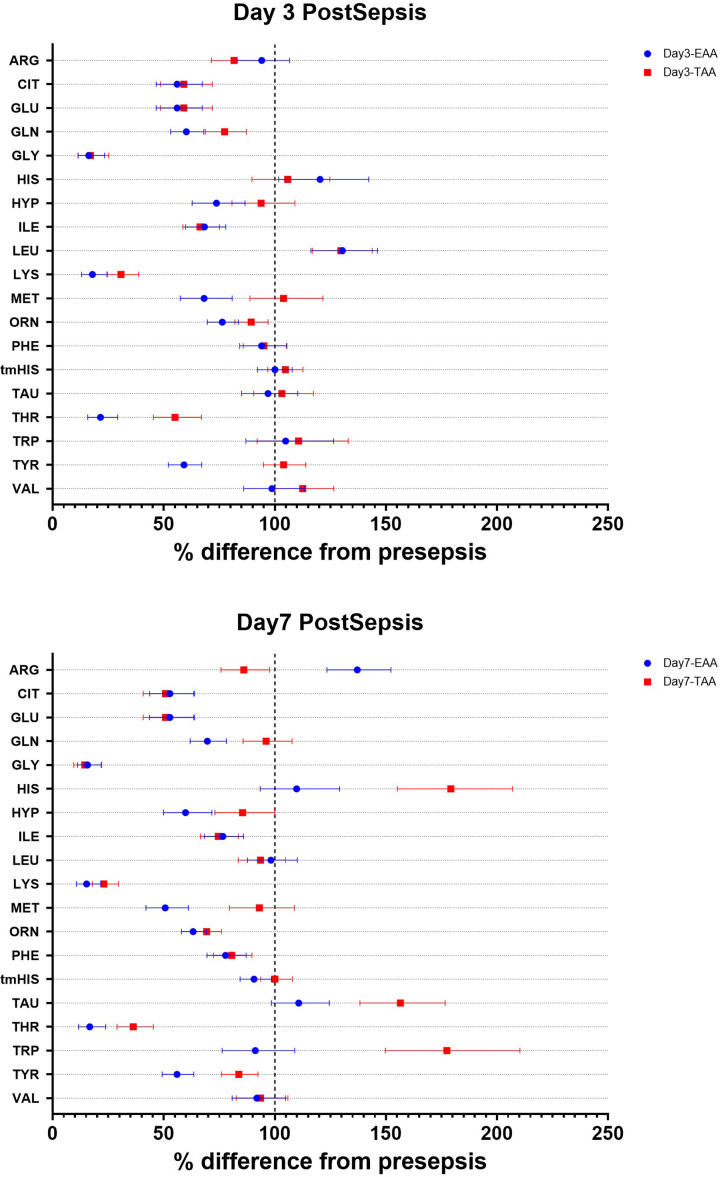
Changes in the Clearance Rate Forest plots showing the changes in clearance rate at day 3 post-sepsis (top panel: A) and day 7 post-sepsis (bottom panel: B). Data are expressed as the percentage difference from presepsis and the 95% CI.

We also calculated the clearance of the amino acids (Figure 4 and Supplementary Table S4). On day 3, reduced clearance was observed in the TAA group for citrulline, glutamate, glutamine, glycine, isoleucine, lysine, ornithine, and threonine, while the clearance of leucine was increased. Compared with the TAA group, the EAA group showed a greater reduction in lysine, methionine, threonine, and tyrosine clearance and a lesser reduction in arginine clearance.

On day 7, clearance remains reduced for many amino acids. In the EAA group, higher clearance of arginine and lower clearance of glutamine, histidine, lysine, methionine, taurine, threonine, tryptophan, and tyrosine were observed compared with the TAA group.

### Compartmental analysis of amino acids

**Figure 5:**
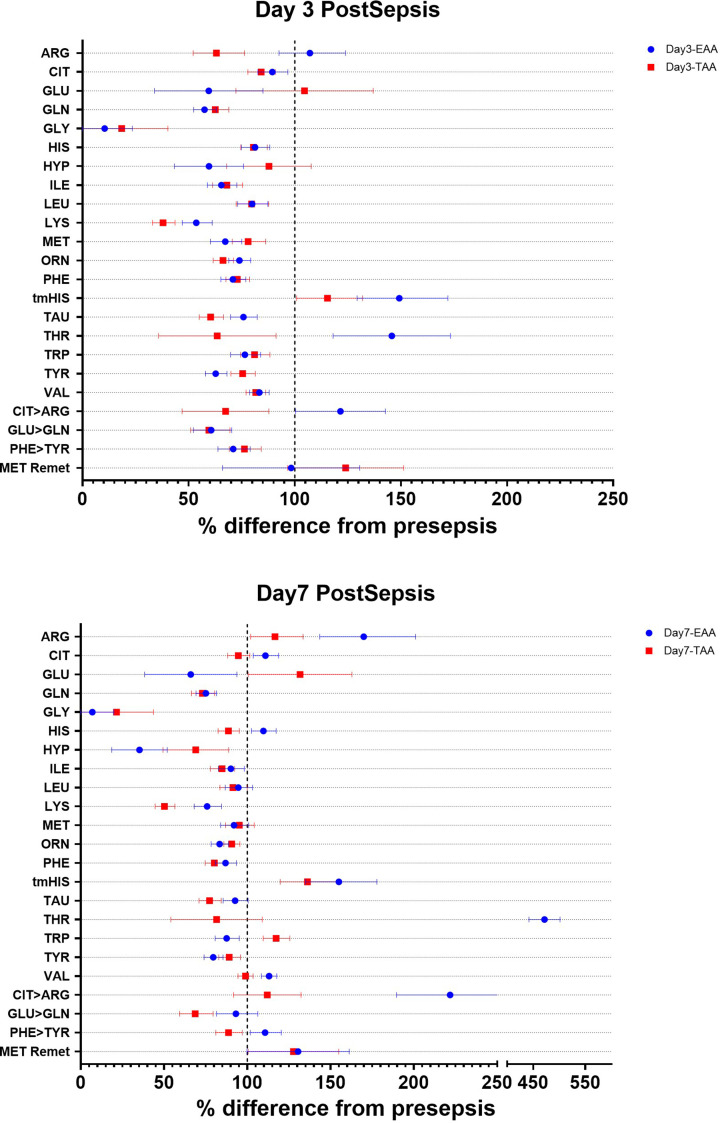
Changes in the Intracellular Production Forest plots showing the changes in intracellular production at day 3 post-sepsis (top panel: A) and day 7 post-sepsis (bottom panel: B). Data are expressed as the percentage difference from presepsis and the 95% CI.

Compartmental analysis was performed to estimate the extracellular and intracellular estimated pool size and intracellular production. The relative changes in intracellular production (Figure 5 and Table 5) were generally comparable with those in the WBP (Figure 3), though with greater variation. Interesting differences in intracellular amino acid production were observed: arginine was lower in the TAA group on day 3, while on day 7 arginine was higher and glutamate lower in the EAA group. Overall, these results suggest that the ratio between the appearance in plasma and intracellularly remained unchanged.

**Table 5:** Whole-body intracellular production of amino acids

Amino acid	*P* ANOVA	Pre-sepsis		Day 3 post-sepsis			Day 7 post-sepsis		
	Group time Group × Time	Group 1	Group 2	EAA	TAA	TAA−EAA	EAA	TAA	TAA−EAA
Arginine	0.0033 <0.001 <0.001	404.4 [347.8, 470.2]	355.8 [304.8, 415.4]	433.1 [374.4, 501.1] *P* = 0.8991	224.5 [185.5, 271.7] *P≤*0.001	−208.7 [−281.7, −135.6] *P≤*0.001	686.8 [580.2, 813.1] *P≤*0.001	415 [362.7, 474.8] *P* = 0.2059	−271.9 [−399.4, −144.3] *P≤*0.001
Citrulline	0.2441 <0.001 <0.001	31 [28.9, 33.3]	30.8 [28.7, 33.1]	27.7 [25.6, 30] *P* = 0.0313	25.9 [24, 28] *P≤*0.001	−1.8 [−4.7, 1.1] *P* = 0.4083	34.4 [32.1, 36.8] *P* = 0.0407	29.2 [27.2, 31.3] *P* = 0.657	−5.2 [−8.3, −2.1] *P* = 0.0022
Glutamate	0.5312 0.1661 0.1946	1019.2 [703.9, 1334.5]	762.1 [388, 1136.2]	606.3 [346.1, 866.4] *P* = 0.1424	797.2 [550.5, 1043.9] *P* = 1	191 [−163.6, 545.5] *P* = 0.4975	673.1 [391.2, 955] *P* = 0.4295	1003.5 [766.6, 1240.4] *P* = 0.8441	330.4 [−37, 697.8] *P* = 0.1498
Glutamine	1 <0.001 1	401.9 [376.8, 428.6]	349.1 [323.3, 377]	231 [210.5, 253.5] *P≤*0.001	218.4 [198.2, 240.7] *P≤*0.001	−12.6 [−39.2, 14.1] *P* = 0.5843	301.9 [278.2, 327.5] *P≤*0.001	254.9 [231.8, 280.3] *P≤*0.001	−46.9 [−80, −13.9] *P* = 0.0106
Glycine	0.0194 <0.001 <0.001	1719.6 [1488.4, 1950.8]	969.7 [722.9, 1216.5]	179.3 [−45.6, 404.2] *P≤*0.001	178.8 [−32.6, 390.1] *P≤*0.001	−0.5 [−290.8, 289.8] *P* = 1	118.7 [−102.7, 340.1] *P≤*0.001	207.8 [−7.4, 423.1] *P≤*0.001	89.1 [−216.9, 395.1] *P* = 0.8135
Histidine	0.4445 <0.001 <0.001	106 [98.9, 113.6]	108.2 [101.5, 115.5]	86.2 [79.3, 93.6] *P≤*0.001	87.2 [80.7, 94.2] *P≤*0.001	1 [−8.3, 10.4] *P* = 0.9713	116.3 [108.6, 124.4] *P* = 0.1384	95.9 [89.3, 103] *P* = 0.0236	−20.4 [−30.7, −10] *P≤*0.001
Hydroxy proline	0.838 <0.001 0.027	34.7 [29.2, 40.1]	27.5 [22, 33]	20.7 [15, 26.3] *P≤*0.001	24.1 [18.7, 29.6] *P* = 0.9096	3.5 [−3.9, 10.8] *P* = 0.5817	12.2 [6.4, 18] *P≤*0.001	19 [13.6, 24.4] *P* = 0.0929	6.8 [−1.1, 14.6] *P* = 0.1769
Isoleucine	0.0814 <0.001 0.1441	181.2 [166.7, 196.9]	164.7 [151.6, 179]	118.5 [106.6, 131.8] *P≤*0.001	112 [101, 124.2] *P≤*0.001	−6.5 [−23.3, 10.2] *P* = 0.6933	163.2 [149.7, 177.9] *P* = 0.1532	139.7 [128.1, 152.3] *P* = 0.0058	−23.5 [−42, −5] *P* = 0.0257
Leucine	1 0.3349 1	301 [278.9, 324.9]	286.6 [263.9, 311.2]	240.5 [220.1, 262.9] *P≤*0.001	228.3 [207.4, 251.3] *P≤*0.001	−12.2 [−38.1, 13.7] *P* = 0.5835	284.7 [260.9, 310.6] *P* = 0.7935	261.9 [239.5, 286.4] *P* = 0.1642	−22.8 [−53.2, 7.7] *P* = 0.2665
Lysine	0.001 <0.001 <0.001	1320.5 [1173.9, 1485.3]	1284.1 [1145.8, 1439.1]	707.9 [621.3, 806.6] *P≤*0.001	487.2 [423.7, 560.1] *P≤*0.001	−220.7 [−335.7, −105.8] *P≤*0.001	1001.6 [899.4, 1115.5] *P≤*0.001	644.7 [572.7, 725.8] *P≤*0.001	−356.9 [−486.2, −227.5] *P≤*0.001
Methionine1	0.0944 <0.001 <0.001	75.2 [69.6, 81.3]	76.3 [70.6, 82.5]	49.1 [44.2, 54.6] *P≤*0.001	52.1 [47.3, 57.4] *P≤*0.001	3 [−3.2, 9.2] *P* = 0.5735	62.3 [56.9, 68.3] *P* = 0.0063	64.1 [58.5, 70.3] *P* = 0.0099	1.8 [−6.3, 9.9] *P* = 0.8838
Methionine3	1 <0.001 0.0012	102.5 [93.8, 112]	98.7 [90.1, 108.1]	68.9 [61.8, 76.9] *P≤*0.001	77 [69.6, 85.2] *P≤*0.001	8.1 [−2.6, 18.8] *P* = 0.2574	94.3 [86.1, 103.3] *P* = 0.2452	93.9 [85.8, 102.8] *P* = 0.7568	−0.4 [−12.5, 11.7] *P* = 0.9976
Ornithine	<0.001 <0.001 0.0336	34 [32.4, 35.8]	35.1 [33.4, 36.8]	25.2 [23.5, 27] *P≤*0.001	23.2 [21.6, 24.9] *P≤*0.001	−2 [−4.3, 0.4] *P* = 0.2034	28.4 [26.6, 30.2] *P≤*0.001	31.8 [30.1, 33.5] *P* = 0.0404	3.4 [0.9, 5.8] *P* = 0.013
Phenylalanine	0.5623 <0.001 <0.001	164.7 [153.2, 177]	165.3 [154.2, 177.1]	116.6 [107.3, 126.6] *P≤*0.001	120.5 [111.7, 130.1] *P≤*0.001	3.9 [−9.4, 17.2] *P* = 0.8079	143.1 [133, 154] *P≤*0.001	132.5 [123.4, 142.3] *P≤*0.001	−10.6 [−24.6, 3.5] *P* = 0.2603
Taurine	0.0255 <0.001 <0.001	52.1 [48.2, 56.2]	51.2 [47.4, 55.3]	39.5 [36.3, 42.9] *P≤*0.001	30.9 [28.1, 34] *P≤*0.001	−8.5 [−11.7, −5.4] *P≤*0.001	48.2 [44.5, 52.3] *P* = 0.1086	39.6 [36.3, 43.1] *P≤*0.001	−8.6 [−12.6, −4.7] *P≤*0.001
Tau-methyl-histidine	0.1126 <0.001 <0.001	0.5 [0.4, 0.5]	0.4 [0.4, 0.5]	0.7 [0.6, 0.8] *P≤*0.001	0.5 [0.5, 0.6] *P* = 0.0394	−0.2 [−0.3, −0.1] *P* = 0.0051	0.7 [0.6, 0.8] *P≤*0.001	0.6 [0.5, 0.7] *P≤*0.001	−0.1 [−0.2, 0] *P* = 0.1411
Threonine	<0.001 <0.001 <0.001	217.2 [159.7, 274.7]	208.4 [150.1, 266.8]	316.4 [256.4, 376.5] *P≤*0.001	132.3 [74.6, 190.1] *P≤*0.001	−184.1 [−264.4, −103.8] *P≤*0.001	1024.3 [959.5, 1089.1] *P≤*0.001	170 [112.8, 227.2] *P* = 0.0164	−854.3 [−940.7, −767.9] *P≤*0.001
Tryptophan	1 <0.001 <0.001	48.5 [44.9, 52.4]	45.4 [42, 49]	37.1 [33.9, 40.7] *P≤*0.001	36.8 [33.8, 40.1] *P≤*0.001	−0.3 [−4.9, 4.3] *P* = 0.9893	42.5 [39.1, 46.1] *P* = 0.0134	53.2 [49.7, 57] *P≤*0.001	10.7 [5.7, 15.7] *P≤*0.001
Tyrosine	1 <0.001 0.9826	160.5 [150.8, 170.9]	147 [137.5, 157.1]	100.8 [92.9, 109.2] *P≤*0.001	110.9 [102.8, 119.7] *P≤*0.001	10.2 [−1.1, 21.4] *P* = 0.1494	127.7 [118.8, 137.2] *P≤*0.001	131 [121.7, 140.9] *P* = 0.003	3.3 [−10.5, 17.1] *P* = 0.8702
Valine	0.0015 <0.001 <0.001	195.3 [186.7, 203.9]	187.1 [178.4, 195.9]	162.8 [153.8, 171.7] *P≤*0.001	152.8 [144.2, 161.4] *P≤*0.001	−10 [−21.7, 1.8] *P* = 0.182	220.8 [211.7, 229.8] *P≤*0.001	185.1 [176.6, 193.6] *P* = 0.9993	−35.7 [−48, −23.3] *P≤*0.001
CIT>ARG	<0.001 <0.001 <0.001	56.6 [44.6, 68.7]	55.8 [44.1, 67.6]	68.8 [56.9, 80.8] *P* = 0.4809	37.6 [26.2, 49.1] *P* = 0.0685	−31.2 [−46.7, −15.7] *P≤*0.001	125.6 [107.4, 143.8] *P≤*0.001	62.5 [51.2, 73.9] *P* = 0.9355	−63 [−84.4, −41.7] *P≤*0.001
GLU>GLN	1 <0.001 <0.001	58.4 [51.5, 66.2]	59.4 [52, 67.8]	35.4 [30.5, 41.1] *P≤*0.001	35.3 [30.3, 41.2] *P≤*0.001	−0.1 [−6, 5.8] *P* = 0.9996	54.4 [47.6, 62.1] *P* = 0.5669	40.8 [35.3, 47.2] *P≤*0.001	−13.6 [−21.4, −5.8] *P* = 0.0012
PHE>TYR	0.1445 <0.001 <0.001	23 [21.1, 25.2]	22 [20.1, 24]	16.4 [14.7, 18.2] *P≤*0.001	16.8 [15.2, 18.5] *P≤*0.001	0.4 [−1.9, 2.8] *P* = 0.9284	25.5 [23.4, 27.7] *P* = 0.1194	19.5 [17.8, 21.3] *P* = 0.0668	−6 [−8.8, −3.2] *P≤*0.001
Methionine ReMethylation	0.4295 0.1722 0.3544	22.6 [15.2, 30.1]	25.3 [18.4, 32.1]	22.2 [14.9, 29.5] *P* = 1	31.3 [24.4, 38.2] *P* = 0.6236	9.1 [0.2, 18] *P* = 0.0876	29.5 [22.6, 36.5] *P* = 0.6088	32.3 [25.4, 39.1] *P* = 0.5116	2.8 [−6.6, 12.1] *P* = 0.8109

Whole-body intracellular productions are mean in μmol/min [95% CI]. Statistics (JASP) by generalized linear mixed model (Family: Gaussian, Link: Identify or Log) with study group, study day, and pig weight as fixed effects variables and pig ID as random effect grouping factor. Post hoc testing *P*-values are corrected by Tukey. Group 1 are the pigs that received EAA feeding, and Group 2 are the pigs that received TAA feeding post-sepsis. *P* ANOVA is the *P*-value of the main effect group.

At day 3 (Supplementary Figure S3 and Supplementary Table S5), the lower extracellular pool size Q_1_ observed for many amino acids was accompanied by higher Q_1_ for glutamate, lysine, and threonine. In the EAA group, Q_1_ was higher for glutamine, histidine, lysine, methionine, tau-methyl histidine, taurine, tyrosine, and valine, but lower for glycine and hydroxyproline.

On day 3 (Supplementary Figure S4 and Supplementary Table S6), the lower estimated Q_2_ was observed for many amino acids and was accompanied by higher Q_2_ for glutamate, lysine, tau-methylhistidine, and threonine. In the EAA group, Q_2_ was lower for glycine but higher for glutamate, histidine, lysine, tau-methyl histidine, threonine, and tyrosine. On day 7, most changes in Q_1_ and Q_2_ were comparable to those on day 3, but the differences between the EAA and TAA groups became much more pronounced.

### Cytokines after sepsis

**Figure 6:**
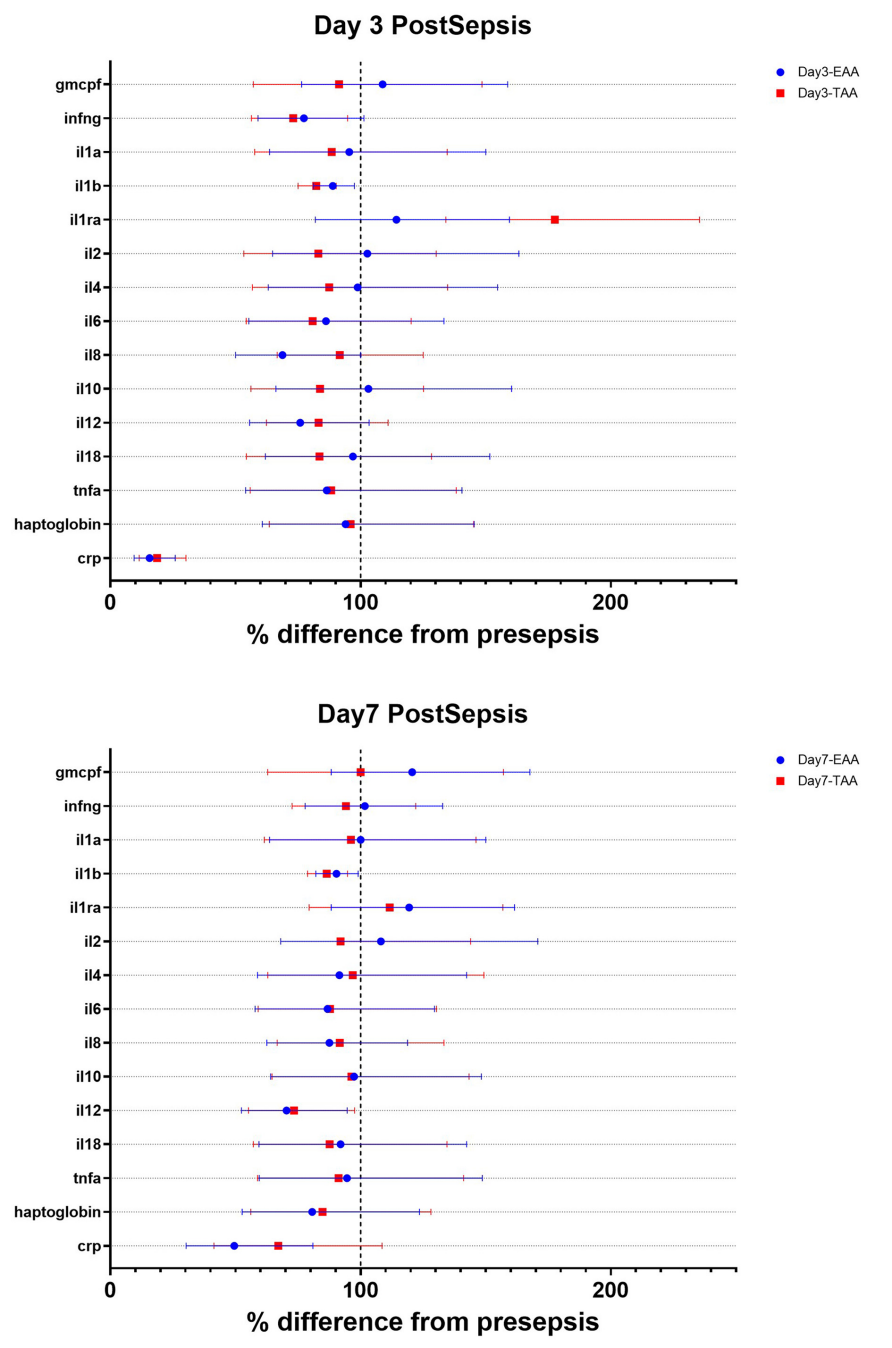
Changes in CRP and Cytokines Forest plots showing the changes in plasma CRP and cytokine concentrations at day 3 post-sepsis (top panel: A) and day 7 post-sepsis (bottom panel: B). Data are expressed as the percentage difference from presepsis and the 95% CI.

Plasma cytokine concentrations were also measured (Figure 6 and Table 6). Both IL-1α and IL-1β were reduced on days 3 and 7 in the EAA group. Interestingly, the plasma CRP concentration was lower in both groups.

**Table 6:** Plasma concentration of cytokines

Amino Acid	*P* ANOVA	Pre-sepsis		Day 3 post-sepsis			Day 7 post-sepsis		
	Group time Group × Time	Group 1	Group 2	EAA	TAA	TAA–EAA	EAA	TAA	TAA–EAA
GMCPF	0.669 0.569 0.537	0.034 [0.024, 0.048]	0.035 [0.02, 0.061]	0.037 [0.026, 0.054] *P* = 0.949	0.032 [0.02, 0.052] *P* = 0.973	−0.005 [−0.024, 0.013] *P* = 0.83	0.041 [0.03, 0.057] *P* = 0.433	0.035 [0.022, 0.055] *P* = 1	−0.006 [−0.026, 0.014] *P* = 0.812
Interferon gamma	0.203 <0.001 0.882	7.281 [5.587, 9.491]	5.112 [3.924, 6.66]	5.63 [4.295, 7.378] *P* = 0.081	3.737 [2.882, 4.847] *P* = 0.023	−1.892 [−3.632, −0.152] *P* = 0.065	7.404 [5.669, 9.67] *P* = 1	4.812 [3.713, 6.238] *P* = 0.994	−2.591 [−4.92, −0.263] *P* = 0.057
IL1a	0.695 0.494 0.605	0.022 [0.014, 0.034]	0.026 [0.017, 0.04]	0.021 [0.014, 0.033] *P* = 1	0.023 [0.015, 0.035] *P* = 0.495	0.002 [−0.012, 0.015] *P* = 0.965	0.022 [0.014, 0.033] *P* = 1	0.025 [0.016, 0.038] *P* = 0.999	0.004 [−0.011, 0.018] *P* = 0.862
IL1b	<0.001 <0.001 0.555	0.207 [0.189, 0.228]	0.288 [0.263, 0.316]	0.184 [0.168, 0.202] *P≤*0.001	0.237 [0.216, 0.26] *P≤*0.001	0.052 [0.047, 0.058] *P≤*0.001	0.187 [0.17, 0.205] *P≤*0.001	0.249 [0.227, 0.273] *P≤*0.001	0.062 [0.056, 0.069] *P≤*0.001
IL1ra	1 <0.001 1	0.802 [0.561, 1.146]	0.673 [0.476, 0.953]	0.917 [0.657, 1.279] *P* = 0.936	1.196 [0.903, 1.586] *P≤*0.001	0.28 [−0.161, 0.72] *P* = 0.3808	0.958 [0.708, 1.295] *P* = 0.8512	0.752 [0.535, 1.056] *P* = 0.9926	−0.206 [−0.589, 0.177] *P* = 0.4988
IL2	0.888 0.431 0.193	0.185 [0.117, 0.295]	0.225 [0.144, 0.353]	0.19 [0.12, 0.302] *P* = 1	0.187 [0.12, 0.293] *P* = 0.187	−0.003 [−0.124, 0.118] *P* = 0.999	0.2 [0.126, 0.316] *P* = 0.942	0.207 [0.133, 0.324] *P* = 0.909	0.008 [−0.123, 0.139] *P* = 0.991
IL4	0.88 0.397 0.204	1.366 [0.873, 2.137]	1.501 [0.971, 2.32]	1.35 [0.862, 2.114] *P* = 1	1.313 [0.852, 2.023] *P* = 0.362	−0.037 [−0.866, 0.791] *P* = 0.995	1.25 [0.803, 1.945] *P* = 0.827	1.454 [0.944, 2.241] *P* = 0.999	0.204 [−0.638, 1.046] *P* = 0.866
IL6	0.009 0.017 0.849	0.159 [0.103, 0.246]	0.188 [0.125, 0.283]	0.137 [0.088, 0.212] *P* = 0.678	0.152 [0.102, 0.226] *P* = 0.346	0.015 [−0.072, 0.102] *P* = 0.931	0.138 [0.092, 0.206] *P* = 0.843	0.165 [0.111, 0.245] *P* = 0.874	0.027 [−0.059, 0.114] *P* = 0.787
IL8	0.519 <0.001 0.221	0.016 [0.011, 0.023]	0.012 [0.009, 0.018]	0.011 [0.008, 0.016] *P* = 0.003	0.011 [0.008, 0.015] *P* = 0.599	0 [−0.006, 0.005] *P* = 0.992	0.014 [0.01, 0.019] *P* = 0.446	0.011 [0.008, 0.016] *P* = 0.939	−0.002 [−0.008, 0.004] *P* = 0.72
IL10	1 1 0.926	0.381 [0.246, 0.591]	0.413 [0.274, 0.621]	0.393 [0.252, 0.611] *P* = 1	0.346 [0.232, 0.517] *P* = 0.345	−0.046 [−0.269, 0.176] *P* = 0.9	0.371 [0.244, 0.565] *P* = 1	0.398 [0.267, 0.592] *P* = 0.999	0.026 [−0.196, 0.248] *P* = 0.967
IL12	0.864 <0.001 0.342	0.771 [0.567, 1.047]	0.71 [0.531, 0.95]	0.585 [0.429, 0.797] *P* = 0.001	0.591 [0.443, 0.788] *P* = 0.036	0.006 [−0.243, 0.254] *P* = 0.999	0.543 [0.404, 0.73] *P≤*0.001	0.521 [0.392, 0.693] *P* = 0.001	−0.022 [−0.237, 0.194] *P* = 0.975
IL18	1 0.085 0.794	1.012 [0.648, 1.579]	1.133 [0.735, 1.746]	0.981 [0.627, 1.535] *P* = 1	0.947 [0.616, 1.454] *P* = 0.354	−0.034 [−0.63, 0.561] *P* = 0.992	0.931 [0.601, 1.441] *P* = 0.961	0.993 [0.647, 1.524] *P* = 0.747	0.063 [−0.528, 0.653] *P* = 0.973
TNFa	1 0.003 0.734	0.037 [0.023, 0.059]	0.034 [0.021, 0.053]	0.032 [0.02, 0.052] *P* = 0.186	0.03 [0.019, 0.047] *P* = 0.435	−0.002 [−0.022, 0.018] *P* = 0.97	0.035 [0.022, 0.055] *P* = 0.914	0.031 [0.02, 0.048] *P* = 0.69	−0.004 [−0.025, 0.016] *P* = 0.902
Haptoglobin	0.891 0.446 0.99	719.4 [468.9, 1103.7]	673.6 [442.5, 1025.5]	676.1 [436.9, 1046] *P* = 0.999	647 [427.9, 978.2] *P* = 1	−29.1 [−420.7, 362.5] *P* = 0.987	580.1 [378.6, 888.9] *P* = 0.759	571.3 [378.2, 863] *P* = 0.901	−8.8 [−350.6, 332.9] *P* = 0.998
CRP	0.189 <0.001 0.563	49.9 [30.3, 82.2]	47.4 [29.1, 77.2]	7.8 [4.7, 13] *P≤*0.001	8.8 [5.5, 14.3] *P≤*0.001	1 [−4.6, 6.6] *P* = 0.923	24.7 [15.1, 40.4] *P* = 0.027	31.8 [19.6, 51.4] * P* = 0.212	7 [−12.4, 26.5] *P* = 0.727

Plasma concentrations are mean in mg/l [95% CI]. Statistics (JASP) by generalized linear mixed model (Family: Gamma, Link: Identify or Log) with study group, study day, and pig weight as fixed effects variables and pig ID as random effect grouping factor. Post hoc testing *P*-values are corrected by Tukey. Group 1 are the pigs that received EAA feeding, and Group 2 are the pigs that received TAA feeding post-sepsis. *P* ANOVA is the *P*-value of the main effect group.

## Discussion

The present study demonstrates that the response to feeding either only essential amino acids or a complete amino acid mixture affects metabolism differently in our sepsis recovery pig model. Our compartmental analysis reveals that the kinetics of several amino acids and the size of the intracellular compartment were either not normalized or were even higher following post-sepsis feeding with either EAA or TAA, suggesting that the nutritional support provided was not optimal.

### Effects of sepsis on amino acid metabolism

We observed a reduction in net protein breakdown after sepsis in the TAA group, a finding consistent with previous observations in humans [15]. We speculate that this reduction is linked to overall body protein catabolism and may be (in)directly connected to autophagy activity as noted in human sepsis studies [38,39]. To compensate for autophagy inactivation, the body increases the ubiquitin-proteasome system in skeletal muscles, which has been shown to worsen sepsis-induced muscle wasting, impair whole-body metabolism, and decrease survival in a mouse model [40]. We previously reported [7] that metabolic changes in pigs are pronounced as early as 6.5 h after sepsis onset. The present study extends this observation, showing that many metabolic changes persist for up to 7 days post-sepsis, with some being independent of the type of nutrition provided. For instance, at day 3 in both intervention groups, plasma levels of arginine, glutamate, leucine, phenylalanine, taurine, and tryptophan were reduced, suggesting these changes are specific to the post-sepsis recovery period. Most of the changes were reversed by day 7, though differences were observed between the dietary interventions. The WBP and intracellular production were affected substantially more following sepsis. At day 3, most amino acid productions were reduced in both intervention groups, except for threonine, suggesting the continuation of overall reduction in metabolism related to sepsis [7]. Another possible explanation for this reduced turnover is that it was caused by intake reduction, as only 50% of the pre-sepsis amino acid intake (54 grams; approximately 2.2 gr/kg BW/day) was provided on day 2 post-sepsis.

The production of essential amino acids was comparable between both intervention groups, but the EAA content in the diet was below 100% from day 1 to day 3. The lower intracellular production observed at day 3 compared with the pre-sepsis period suggests that neither intervention meal provided sufficient essential amino acids. On day 7, food intake was restored to 100% (108 grams of amino acids; approximately 4.4 g/kg BW/day) and maintained for 4 days. However, the intracellular production of several non-essential amino acids, particularly glutamine and glycine, was still lower. These persistent changes must be related to the sepsis event that occurred 7 days prior. Thus, even though the plasma concentrations of glutamate and glutamine had normalized, their production remained significantly reduced. Our results in the pig are in contrast with what we previously observed in humans with sepsis [12,15].

### Metabolic consequences of providing only essential amino acids post-sepsis

We intentionally provided only essential amino acids in the EAA group to specifically observe the effects on non-essential amino acid metabolism. Overall, the plasma concentration, and particularly, the production of most amino acids was not significantly different between the intervention groups, with the exception of lysine. This suggests that the EAA dose (54 g/day) had an effect on metabolism comparable to the TAA mixture (20 g EAA and 34 g NEAA), implying a similar quality of the nutrition between the groups. By day 7, most essential amino acid productions were normalized in the TAA group, while in the EAA group, they were at 10%–500% levels, suggesting the dosage of some amino acids exceeded their requirements.

The question remains whether the conversion of the essential amino acids to the non-essential amino acids was sufficient in the EAA group. Although the TAA diet contained glutamate, glutamine, and glycine, the reduction in the production of these amino acids was comparable in both the TAA and EAA groups. This result suggests that the conversion was impaired post-sepsis, and the impairment was not differently affected by the two diets. Therefore, further exploration is needed to determine whether increased supplementation with glutamine, glutamate, or glycine is necessary to compensate for this reduced production. Arginine and citrulline production normalized in both intervention groups by day 7, indicating that pigs could convert sufficient amounts of citrulline to maintain arginine levels. However, this normalization in the EAA group came with a significant metabolic burden, as cells were required to double the *de novo* arginine production rate compared with the TAA group.

Despite nutritional supplementation, the clearance of several amino acids was reduced, suggesting either a persistently lower demand or adequate provision of nutrients. For example, in the TAA group, arginine was provided, and its clearance was comparable to pre-sepsis levels. In contrast, in the EAA group (where no arginine was provided), clearance was increased by 40%. A similar pattern of clearance was observed for histidine, methionine, tryptophan, threonine, and tyrosine in the EAA compared with the TAA group. It remains unclear whether clearance measurements accurately estimate actual requirements in pigs, but if they do, the amino acid profiles in the EAA or TAA group did not align with the needs of the rehabilitation period.

Pigs are particularly sensitive to reduced lysine intake [41]. After sepsis, we observed a persistent reduction in the intracellular lysine pool and very low clearance rates [7], which were comparable between the EAA and TAA groups. In contrast, plasma pool concentration and WBP of lysine were increased at days 3 and 7 post-sepsis, with a greater increase in the EAA group. These contradictory observations may be related to a possible problem in lysine transport into the cells that warrants further investigation.

### The metabolic changes in taurine

Taurine is a non-protein amino acid. Although we previously observed no change in its plasma concentration during sepsis, we found an increase in its extracellular and intracellular pool, intracellular production, and clearance [7]. Post-sepsis, we found reduced plasma taurine concentrations and lower extracellular and intracellular pools and lower intracellular production, even though clearance was unchanged on day 3 but higher on day 7. Others have also reported reduced plasma taurine after sepsis [42]. Given taurine’s role in muscle function due to its antioxidant and anti-inflammatory properties [42,43], our data supports the notion that taurine production during sepsis is insufficient. However, evidence for supplementation in critically ill adults is inconclusive, as one study in acute lung injury patients who received taurine-enriched enteral nutrition, as compared with other immune-enhancing compounds and antioxidants, showed negative outcomes, including a decrease in ventilator-free days, more days with organ failure, and a trend toward increased mortality [43]. As it remains unclear whether taurine was the sole cause of these negative outcomes [43], large randomized controlled trials are needed to specifically address the impact of taurine supplementation in critically ill adults [43].

### The metabolic changes in hydroxyproline and tau-methylhistidine

Tau-methylhistidine (in myofibrillar protein) and hydroxyproline (in collagen protein) are posttranslationally modified amino acids [11], and their increased release indicates a higher rate of protein breakdown of these specific proteins. Since these proteins constitute a large part of body proteins [11,44] but have a small turnover rate, any increase in their breakdown rate is substantial. We observed a significant increase (20%–25%) in tau-methylhistidine plasma concentration and production 3 days after sepsis, which was higher following the EAA intervention. This indicates increased muscle protein breakdown after sepsis. This accelerated proteolysis activation is likely driven by the systemic activation of the ubiquitin-proteasome system and autophagy-lysosome pathways, which are known to be up-regulated by pro-inflammatory cytokines and glucocorticoids during sepsis to degrade contractile proteins [45]. Given that muscle protein synthesis is often reduced in sepsis [46], we believe this is predominantly protein breakdown. This increased muscle breakdown could well account for the changes in muscle function that we observed. Nevertheless, the breakdown of collagen protein was reduced on days 3 and 7 after sepsis, which is in contrast with the increased breakdown during sepsis [7]. This reduction aligns with studies showing that collagen protein synthesis is suppressed during and after sepsis [47]. Moreover, hydroxyproline reduction during sepsis is associated with an increased hydroxyproline clearance [7], which could possibly be explained by higher activity of hydroxyproline degradation enzymes, such as hydroxyproline dehydrogenase 2 (PRODH2) [48].

### Was the nutritional composition of the amino acid feedings optimal?

Our study on the changes in metabolism after sepsis showed a clear difference when feeding only EAA versus a combination of all amino acids (TAA), which matches the composition of muscle tissue. Since the pigs received a substantial amount of amino acids in their food up to their pre-sepsis intake, we do not believe that insufficient total amino acid supply was the primary issue. Both the EAA and TAA groups showed plasma concentrations that were higher than pre-sepsis for most amino acids. However, the composition of the provided amino acid mixtures is likely the point of concern. For successful protein synthesis, the provided amino acids must match the composition of the tissues that need to regenerate. The TAA was composed to match muscle amino acid composition. We observed that the production of EAA was adequate in the TAA group but that the production of NEAA, which is also vital for protein synthesis, showed major deficiencies. Specifically, the production of glutamine and glycine was substantially reduced at day 7, suggesting that the amount provided in the TAA group was insufficient to support protein synthesis. Production of glycine, as a major component of collagen protein [44], was substantially reduced, which could be directly linked to the lower collagen protein turnover (both protein synthesis and breakdown) observed. Glutamate and glutamine are major components of all body protein (up to 15% of all amino acids) [34], and their deficiency could therefore negatively impact overall protein synthesis. As our measurements of WBP and intracellular production were only conducted in the postabsorptive condition, studies performed during feeding can definitely answer whether protein synthesis response to nutrition is reduced.

### What is the benefit of performing compartmental analysis?

We have previously shown that relying solely on plasma concentration of amino acids does not provide sufficient insight into the complex changes in intracellular amino acid metabolism during sepsis, making it necessary to measure the intracellular production [7]. In the present study, compartmental analysis proved crucial for better understanding the changes in intracellular amino acid metabolism. Our approach, which involved using pulse amino acid tracers combined with measurements during feeding [49], is designed to further enhance our understanding of amino acid metabolism after sepsis and the response to nutritional intervention. In our opinion, combining our translational pig sepsis model with postabsorptive and feeding measurements will enable us to evaluate more precisely various nutritional interventions with the aim of reducing muscle function loss and normalizing overall metabolism.

## Conclusions

In the sepsis-recovery pig model, intake of EAA alone was less adequate in restoring overall amino acid metabolism and resulted in higher net protein breakdown compared with TAA intake. Providing either essential amino acids alone or a complete amino acid mixture failed to fully normalize the intracellular production of key non-essential amino acids, particularly glutamine and glycine, suggesting a persistent deficiency in NEAAs after sepsis. These results highlight the need for a reevaluation of the optimal amino acid composition to support metabolic recovery after sepsis.

## Clinical perspectives

- Recovery from sepsis is characterized by profound disturbances in amino acid metabolism and protein turnover, making optimal amino acid nutritional support critical for restoring anabolic balance.
- We demonstrated in a pig model that supplementation with essential amino acids alone, compared with a balanced mixture of all amino acids, resulted in a higher protein breakdown and that both nutritional interventions failed to fully normalize intracellular production of amino acids.
- These findings suggest that current post-sepsis nutritional strategies require further evaluation, emphasizing the vital importance of both essential and non-essential amino acids. Translating this approach to human rehabilitation after sepsis may lead to improved metabolic recovery outcomes.

## Supplementary Material

Supplementary Figures S1-S4 and Tables S1-S6

## Data Availability

Data described in the manuscript, code book, and analytic code will be made available upon request, pending approval of the principal investigator (nep.deutz@ctral.org).

## Open Access

Open access for this article was enabled by the participation of Texas A&M University in an all-inclusive Read & Publish agreement with Portland Press and the Biochemical Society.

## Abbreviations

AA: amino acids
CI: confidence interval
CRP: C-reactive protein
EAA: essential amino acids
Fmoc: 9-fluorenylmethoxycarbonyl
LNAA: large neutral amino acids
NEAA: non-essential amino acids
TAA: total amino acids
TCA: trichloroacetic acid solution
TTR: tracer-to-tracee ratio
WBP: whole-body production
