# Supplementary material for: The metabolic effects of post-sepsis feeding of meals with only essential amino acids
Source: Clin Sci (Lond). 2026 Jun 1;140(6):1175–96. doi: 10.1042/CS20250414 (PMC13226642; doi:10.1042/CS20250414)
Supplement: Supplementary Figures S1-S4 and Tables S1-S6 [file CS-2025-0414_supp.pdf]

Figure 1 (Supplemental) - Metabolic Test Day.

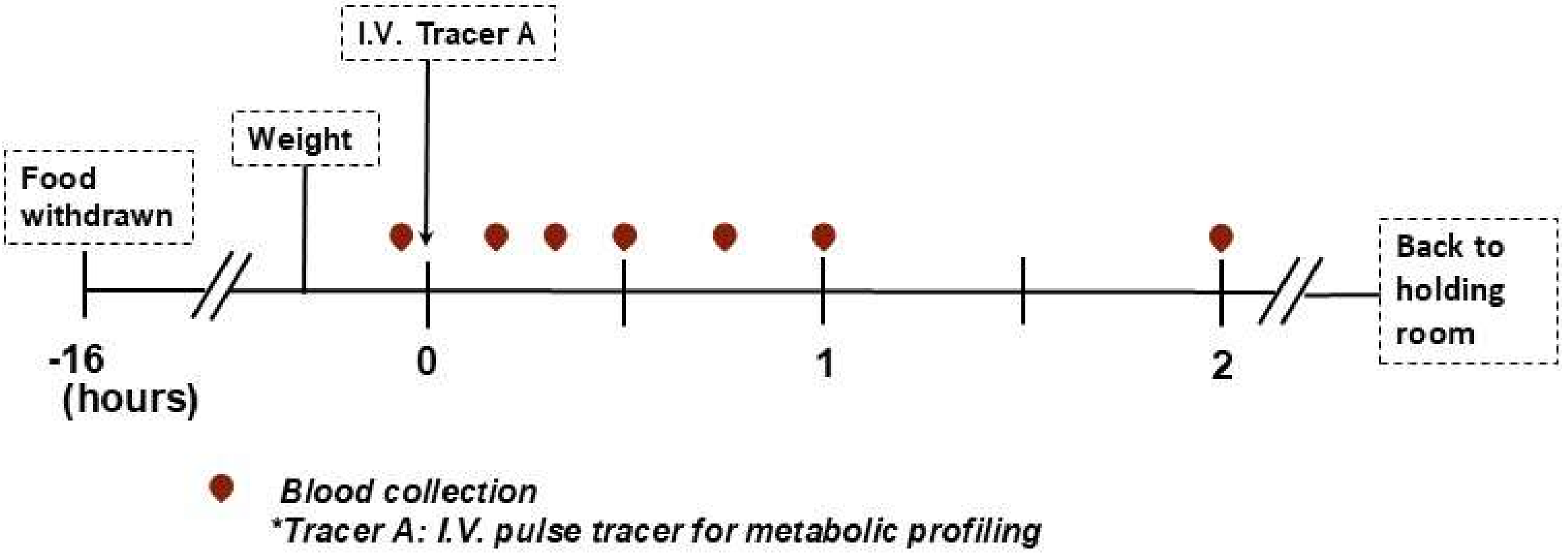

Flow diagram of a metabolic test day

Figure 2 (Supplemental)

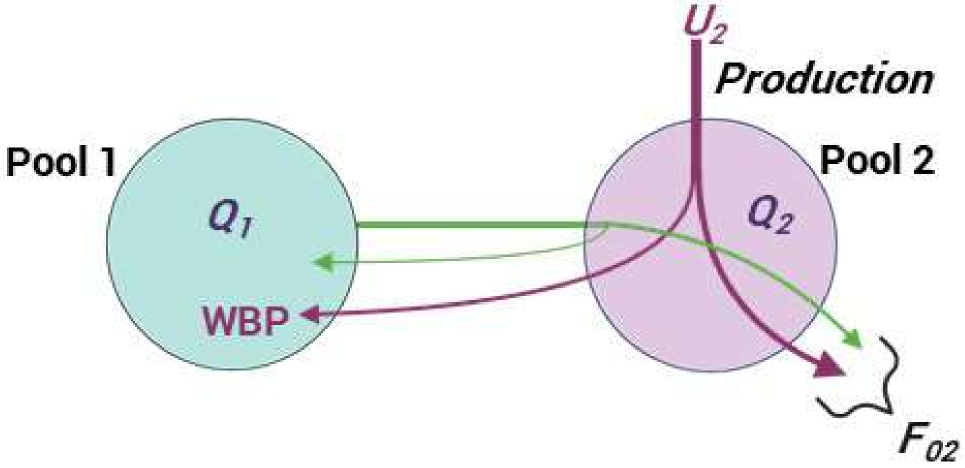

The fluxes between pool 1 (Q_1: extracellular) and pool 2 (Q_2: intracellular).

Figure 3 (Supplemental) – Q1

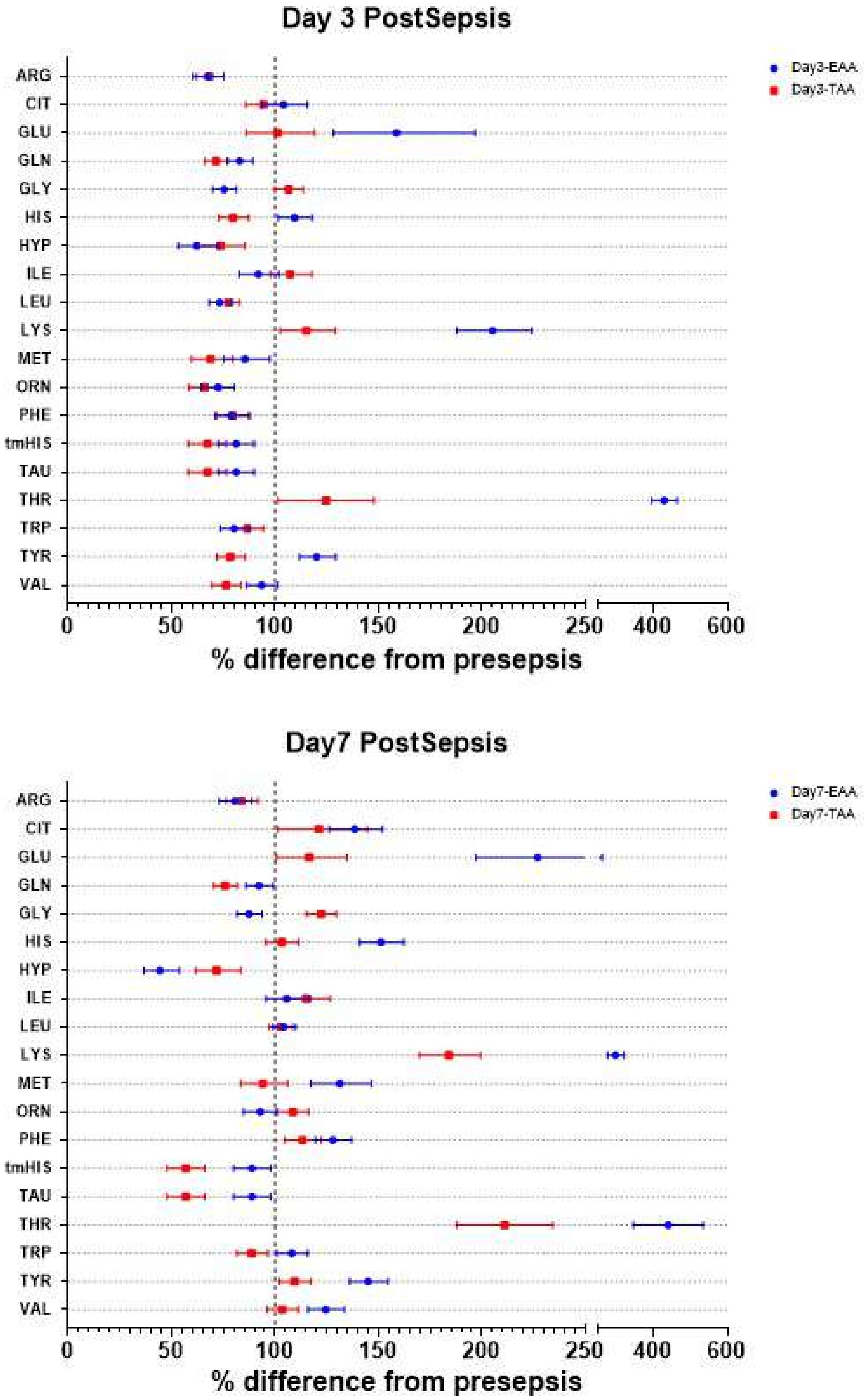

Forest plots showing the changes in extracellular pool size (Q_1) at day 3 post-sepsis (top panel: A) and day 7 post-sepsis (bottom panel: B). Data are expressed as the percentage difference from presepsis and the 95% confidence interval.

Figure 4 (Supplemental) – Q2

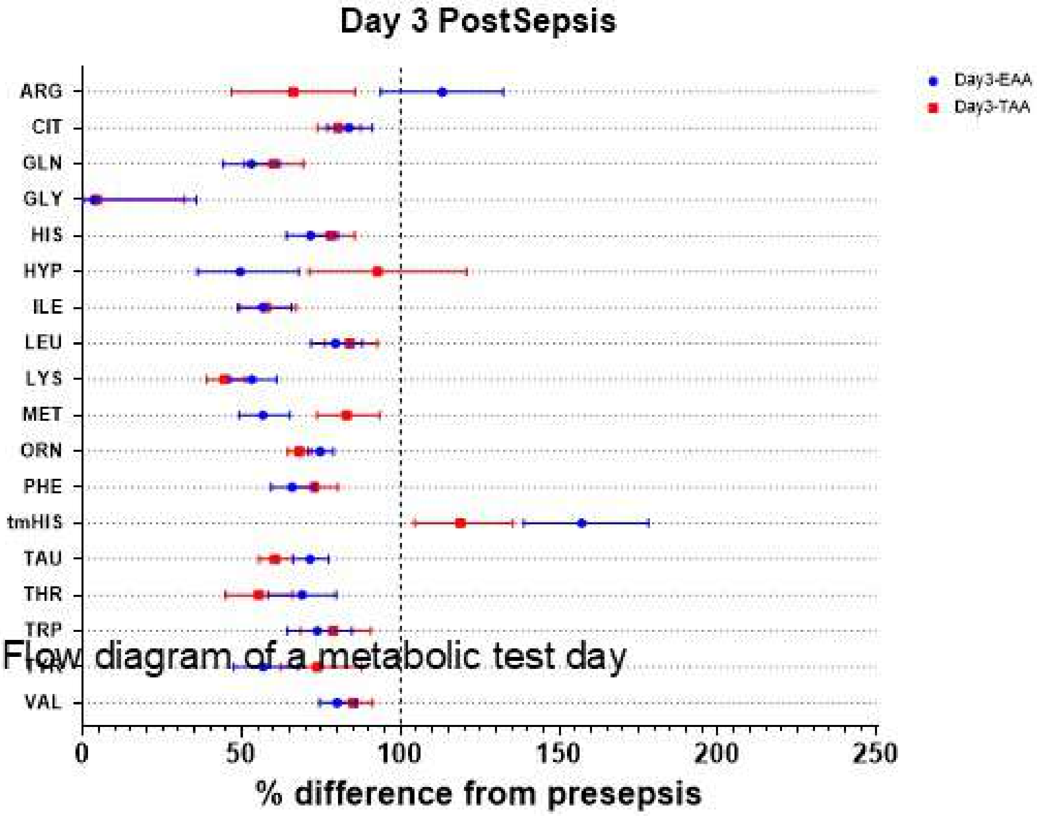

Flow diagram of a metabolic test day

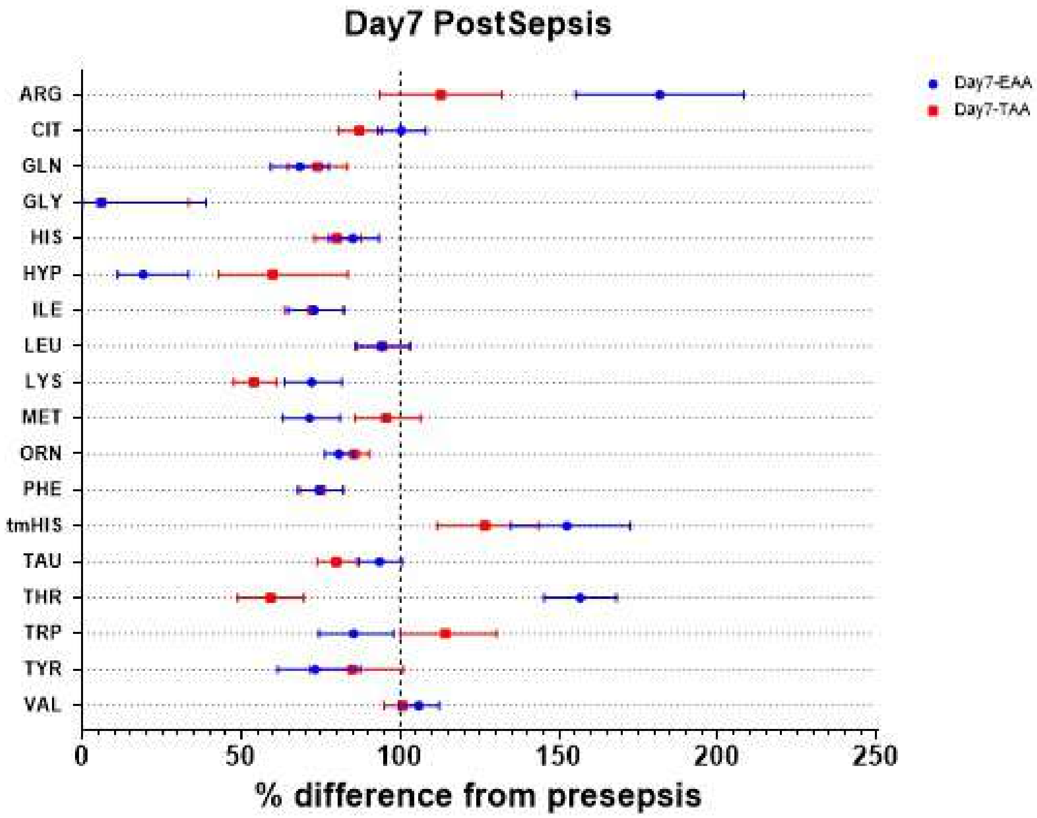

Forest plots showing the changes in intracellular pool size (Q_2) at day 3 post-sepsis (top panel: A) and day 7 post-sepsis (bottom panel: B). Data are expressed as the percentage difference from presepsis and the 95% confidence interval.

Supplemental Table 1: Nutrients in Pig Food daily and Supplements (TAA/EAA).

Macro and Micronutrients	Food day 1 g/day (25%)		Food day 2 g/day (50%)		Food day 3 g/day (75%)		Food day 4-7 g/day (100%)	
Kcal	786.3		1572.5		2358.8		3145	
Maltodextrine (g)	112.8		225.5		338.3		451	
Xanthum Gum (g)	0.3		0.7		1.0		1.3	
Inulin (g)	1.3		2.5		3.8		5	
OliveOil	25.3		50.5		75.8		101	
KPO4	3.6		7.2		10.8		14.4	
MgSO4	0.8		1.6		2.4		3.2	
CaCl2	0.9		1.7		2.6		3.4	
NaCl	3.5		7.0		10.5		14	
Vitamins (ml)	0.3		0.7		1.0		1.3	
Amino acids (g)	27.0		54.0		81.0		108	
Amino acids	TAA (g/day)	EAA (g/day)	TAA (g/day)	EAA (g/day)	TAA (g/day)	EAA (g/day)	TAA (g/day)	EAA (g/day)
Histidine	1.2	3.3	2.4	6.6	3.6	9.9	4.87	13.18
Isoleucine	0.9	2.6	1.9	5.1	2.8	7.7	3.78	10.27
Leucine	1.7	4.7	3.5	9.3	5.2	14.0	6.92	18.69
Lysine	2.3	6.3	4.6	12.5	6.9	18.8	9.23	25.03
Methionine	0.5	1.3	0.9	2.5	1.4	3.8	1.85	5.02
Phenylalanine	0.9	2.5	1.8	5.0	2.8	7.5	3.69	10.00
Threonine	1.0	2.6	2.0	5.3	2.9	7.9	3.90	10.53
Tryptophan	0.3	0.7	0.5	1.5	0.8	2.2	1.05	2.91

Valine	1.1	3.1	2.3	6.2	3.4	9.3	4.57	12.38
Arginine	1.6	0.0	3.2	0.0	4.8	0.0	6.33	0.00
Alanine	1.6	0.0	3.2	0.0	4.8	0.0	6.42	0.00
Aspartic Acid	0.8	0.0	1.5	0.0	2.3	0.0	3.02	0.00
Asparagine	2.2	0.0	4.3	0.0	6.5	0.0	8.64	0.00
Cystine	0.3	0.0	0.7	0.0	1.0	0.0	1.38	0.00
Glutamate	3.3	0.0	6.6	0.0	10.0	0.0	13.30	0.00
Glutamine	1.2	0.0	2.4	0.0	3.7	0.0	4.87	0.00
Glycine	2.2	0.0	4.4	0.0	6.6	0.0	8.85	0.00
Proline	2.2	0.0	4.3	0.0	6.5	0.0	8.60	0.00
Serine	0.9	0.0	1.9	0.0	2.8	0.0	3.78	0.00
Tyrosine	0.7	0.0	1.5	0.0	2.2	0.0	2.94	0.00
Essential Amino Acids	*10.0*	*27.0*	*19.9*	*54.0*	*29.9*	*81.0*	*39.9*	*108.0*
Non-Essential Amino Acids	*17.0*	*0.0*	*34.1*	*0.0*	*51.1*	*0.0*	*68.1*	*0.0*

Intra-gastric nutritional intervention was provided as 2 meals/day

Supplemental Table 2: Formula used to estimate the compartment size and fluxes between the compartments

Formula for compartmental calculations	Equation in excel	Equation in GraphPad Prism (Edit equation/Transform to Report)
$WBP = \frac{1}{a/k1 + b/k2}$	1/(a/k1+b/k2)	1/(a/k1+b/k2)
$Q_1 = Q_{EC} = \frac{1}{a+b}$	1/sum(a,b)	(1/(a+b))
$k_{21} = \frac{a \times k_1 + b \times k_2}{a+b}$	sum(a*k1,b*k2)/ sum(a,b)	((a*k1+b*k2)/(a+b))
$k_{12} = \frac{a \times b \times (k_1 - k_2)^2}{(a+b)(a \times k_1 + b \times k_2)}$	(a*b*(k1-k2)^2)/ (sum(a,b)*sum(a*k1,b*k2))	((a*b*(k1-k2)^2)/((a+b)*(a*k1+b*k2)))
$k_{02} = \frac{b \times k_1 + a \times k_2}{a+b} - k_{12}$	(sum(b*k1,a*k2)/ sum(a,b)) - k12	(((b*k1+a*k2)/(a+b)) - (a*b*(k1-k2)^2)/((a+b)*(a*k1+b*k2)))
$F_{21} = F_{12} \Rightarrow k_{21} \times Q_1 = k_{12} \times Q_2$	k21*Q1= k12*Q2	((a*k1+b*k2)/(a+b)) * (1/(a+b))
$Q_2 = \frac{k_{21}}{k_{12}} \times Q_1$	Q1*(k21/k12)	((1/(a+b))*((a*k1+b*k2) / (a+b))/((a*b*(k1- k2)^2)/((a+b)*(a*k1+b*k2))))
$F_{02} = U_2 = k_{02} \times Q_2$	U2 = k02*Q2	(((b*k1+a*k2)/(a+b)) - (a*b*(k1- k2)^2)/((a+b)*(a*k1+b*k2)))*((1/(a+b))*((a*k1+b*k2) /

		(a+b))/((a*b*(k1-k2)^2)/((a+b)*(a*k1+b*k2))))

Supplemental Table 3: Whole Body Production of amino acids

Amino Acid	P ANOVA	Presepsis		Day 3			Day 7		
	Group<br>Time<br>Group*Time	Group1	Group2	EAA	TAA	TAA-EAA	EAA	TAA	TAA-EAA
Arginine	1<br>< .001<br>0.0014	123.9<br>[114.3, 134.3]	110.3<br>[101.7, 119.6]	93.8<br>[85.4, 103]<br>p=< .001	74.8<br>[67.8, 82.5]<br>p=< .001	-19<br>[-30.4, -7.5]<br>p=0.0023	127.9<br>[118, 138.7]<br>p=0.9609	112.5<br>[104.2, 121.5]<br>p=0.997	-15.4<br>[-28.9, -1.8]<br>p=0.0523
Citrulline	0.2959<br>< .001<br>< .001	15.6<br>[14.4, 16.8]	15.4<br>[14.3, 16.6]	14.8<br>[13.6, 16]<br>p=0.7444	13.6<br>[12.5, 14.7]<br>p=0.0195	-1.2<br>[-2.8, 0.4]<br>p=0.2663	18.9<br>[17.6, 20.3]<br>p=< .001	15.6<br>[14.5, 16.8]<br>p=0.9992	-3.3<br>[-5.1, -1.5]<br>p=< .001
Glutamate	< .001<br>< .001<br>0.3557	729.9<br>[659.4, 808]	647.2<br>[585.3, 715.7]	350.9<br>[309.1, 398.4]<br>p=< .001	312.6<br>[275.3, 355]<br>p=< .001	-38.3<br>[-98, 21.3]<br>p=0.3728	304.7<br>[261.4, 355.2]<br>p=< .001	325.5<br>[286.6, 369.8]<br>p=< .001	20.8<br>[-42.4, 84]<br>p=0.7686
Glutamine	1<br>< .001<br>< .001	212.9<br>[202.7, 223.7]	215.6<br>[204.7, 227]	140.7<br>[133, 149]<br>p=< .001	130.8<br>[123.1, 138.9]<br>p=< .001	-10<br>[-18.2, -1.8]<br>p=0.0345	165.8<br>[156.8, 175.4]<br>p=< .001	157.1<br>[148.2, 166.6]<br>p=< .001	-8.7<br>[-19, 1.6]<br>p=0.188
Glycine	1<br>< .001<br>0.1873	495.9<br>[429.3, 572.9]	422.8<br>[363.8, 491.2]	106.6<br>[83.3, 136.4]<br>p=< .001	111.5<br>[88.3, 140.7]<br>p=< .001	4.9<br>[-25.9, 35.6]<br>p=0.9403	92.6<br>[69, 124.3]<br>p=< .001	101.5<br>[80.2, 128.5]<br>p=< .001	8.9<br>[-24, 41.7]<br>p=0.8365
Histidine	1<br>< .001<br>< .001	45<br>[42.4, 47.8]	42.8<br>[40.2, 45.6]	40.9<br>[38.3, 43.6]<br>p=0.0086	33.7<br>[31.5, 36.1]<br>p=< .001	-7.2<br>[-10.2, -4.2]<br>p=< .001	56.3<br>[52.9, 60]<br>p=< .001	39.8<br>[37.3, 42.4]<br>p=0.0892	-16.6<br>[-20.6, -12.6]<br>p=< .001
Hydroxy proline	1<br>< .001<br>< .001	19.4<br>[17, 22.2]	15.1<br>[13.1, 17.5]	11.5<br>[9.8, 13.6]<br>p=< .001	12.9<br>[11.1, 15]<br>p=0.1575	1.4<br>[-1.2, 4]<br>p=0.5031	5.9<br>[4.3, 8]<br>p=< .001	11<br>[9.4, 13]<br>p=< .001	5.2<br>[2.6, 7.7]<br>p=< .001
Isoleucine	0.0024<br>< .001<br>0.2712	69.9<br>[66, 74]	66<br>[62, 70.2]	53.4<br>[50, 57.1]<br>p=< .001	53.2<br>[49.6, 57]<br>p=< .001	-0.2<br>[-4.5, 4]<br>p=0.9919	67.8<br>[63.7, 72.1]<br>p=0.8969	63.1<br>[59.1, 67.5]<br>p=0.7187	-4.6<br>[-9.9, 0.6]<br>p=0.1613
Leucine	0.1027<br>< .001<br>0.0159	117<br>[111.4, 122.8]	114.2<br>[108.8, 119.9]	92.3<br>[87.3, 97.5]<br>p=< .001	87.9<br>[83.3, 92.8]<br>p=< .001	-4.3<br>[-11.3, 2.7]<br>p=0.4018	121.8<br>[116.1, 127.7]<br>p=0.3704	107.4<br>[102.2, 112.8]<br>p=0.0552	-14.4<br>[-22.5, -6.3]<br>p=< .001
Lysine	< .001<br>< .001<br>< .001	291.9<br>[269.3, 316.3]	274.4<br>[257.9, 291.9]	354.5<br>[331.1, 379.5]<br>p=< .001	209.5<br>[189.8, 231.3]<br>p=< .001	-145<br>[-175.8, -114.2]<br>p=< .001	477.4<br>[452.9, 503.3]<br>p=< .001	306.7<br>[286.4, 328.4]<br>p=0.0754	-170.7<br>[-203.6, -137.8]<br>p=< .001
Methionine1	0.0492<br>< .001<br>0.0392	30<br>[28.4, 31.7]	29.1<br>[27.5, 30.8]	23.3<br>[21.7, 25]<br>p=< .001	20.9<br>[19.3, 22.6]<br>p=< .001	-2.4<br>[-4.7, -0.1]<br>p=0.0745	32<br>[30.2, 33.8]<br>p=0.4742	27.3<br>[25.7, 29.1]<br>p=0.6003	-4.7<br>[-7.1, -2.2]<br>p=< .001

Methionine3	0.1491<br>< .001<br>< .001	36.4<br>[33.8, 39.2]	35<br>[32.4, 37.8]	28.3<br>[26.1, 30.8]<br>p=< .001	26.1<br>[23.9, 28.4]<br>p=< .001	-2.3<br>[-5.5, 1]<br>p=0.3155	40.3<br>[37.5, 43.3]<br>p=0.0029	33.5<br>[31.1, 36.1]<br>p=0.6968	-6.7<br>[-10.6, -2.9]<br>p=0.0011
Ornithine	0.2699<br>< .001<br>0.0078	15.8<br>[14.9, 16.7]	16.2<br>[15.3, 17.1]	11.6<br>[10.8, 12.6]<br>p=< .001	10.5<br>[9.6, 11.4]<br>p=< .001	-1.1<br>[-2.4, 0.1]<br>p=0.1318	13.7<br>[12.8, 14.6]<br>p=0.0072	15.8<br>[15, 16.7]<br>p=0.9904	2.1<br>[0.9, 3.4]<br>p=0.0017
Phenylalanine	0.1593<br>< .001<br>< .001	56.7<br>[53.6, 60]	54.4<br>[51.5, 57.5]	43.6<br>[40.8, 46.5]<br>p=< .001	40.9<br>[38.5, 43.6]<br>p=< .001	-2.6<br>[-6.4, 1.1]<br>p=0.3116	57.6<br>[54.5, 60.9]<br>p=0.9907	51.3<br>[48.5, 54.2]<br>p=0.1644	-6.3<br>[-10.6, -2.1]<br>p=0.0069
Taurine	< .001<br>< .001<br>< .001	27.1<br>[25.5, 28.8]	26.5<br>[24.9, 28.2]	20.6<br>[19.3, 22.1]<br>p=< .001	16.9<br>[15.6, 18.2]<br>p=< .001	-3.7<br>[-5.2, -2.2]<br>p=< .001	24.5<br>[22.9, 26.1]<br>p=0.0041	17.8<br>[16.5, 19.2]<br>p=< .001	-6.7<br>[-8.5, -4.9]<br>p=< .001
Tau-methyl-<br>histidine	0.1139<br>< .001<br>0.001	0.4<br>[0.3, 0.4]	0.4<br>[0.3, 0.4]	0.6<br>[0.5, 0.7]<br>p=< .001	0.4<br>[0.4, 0.5]<br>p=0.0361	-0.1<br>[-0.2, -0.1]<br>p=0.0056	0.6<br>[0.5, 0.7]<br>p=< .001	0.5<br>[0.4, 0.6]<br>p=< .001	-0.1<br>[-0.2, 0]<br>p=0.1357
Threonine	< .001<br>< .001<br>< .001	82<br>[62.8, 101.1]	80.2<br>[60.7, 99.7]	177.2<br>[157.2, 197.2]<br>p=< .001	68.1<br>[48.9, 87.3]<br>p=0.9146	-109.1<br>[-135.8, -82.4]<br>p=< .001	474.3<br>[454, 494.7]<br>p=< .001	94.9<br>[75.9, 114]<br>p=0.8282	-379.4<br>[-407.2, -351.5]<br>p=< .001
Tryptophan	0.2085<br>< .001<br>1	18.3<br>[17.5, 19.3]	17.3<br>[16.4, 18.2]	14.2<br>[13.4, 15.1]<br>p=< .001	14<br>[13.3, 14.9]<br>p=< .001	-0.2<br>[-1.3, 1]<br>p=0.9565	18.1<br>[17.3, 19.1]<br>p=0.9992	16.6<br>[15.8, 17.5]<br>p=0.7591	-1.5<br>[-2.7, -0.3]<br>p=0.0322
Tyrosine	1<br>< .001<br>0.0034	59<br>[56.1, 62]	57.7<br>[54.9, 60.6]	49.4<br>[46.7, 52.2]<br>p=< .001	43<br>[40.6, 45.5]<br>p=< .001	-6.4<br>[-10, -2.8]<br>p=< .001	58.6<br>[55.8, 61.7]<br>p=1	55.3<br>[52.6, 58.1]<br>p=0.4462	-3.3<br>[-7.3, 0.7]<br>p=0.2023
Valine	< .001<br>< .001<br>< .001	97.7<br>[93.6, 101.9]	92.7<br>[88.8, 96.7]	80.9<br>[77.1, 84.9]<br>p=< .001	71.9<br>[68.5, 75.4]<br>p=< .001	-9<br>[-14, -4]<br>p=< .001	113.6<br>[109.2, 118.1]<br>p=< .001	92.5<br>[88.8, 96.4]<br>p=1	-21<br>[-26.9, -15.2]<br>p=< .001
CIT>ARG	< .001<br>< .001<br>< .001	17.1<br>[15.3, 19.1]	17.1<br>[15.3, 19]	14.4<br>[12.7, 16.4]<br>p=0.0467	11.1<br>[9.6, 12.7]<br>p=< .001	-3.4<br>[-5.7, -1.1]<br>p=0.0071	25.5<br>[23.2, 28.1]<br>p=< .001	16.3<br>[14.6, 18.1]<br>p=0.9659	-9.2<br>[-12.2, -6.3]<br>p=< .001
GLU>GLN	0.0049<br>< .001<br>< .001	29.5<br>[27, 32.2]	27.8<br>[25.3, 30.5]	20.6<br>[18.5, 23]<br>p=< .001	17.6<br>[15.7, 19.7]<br>p=< .001	-3.1<br>[-5.8, -0.3]<br>p=0.0572	32<br>[29.2, 35]<br>p=0.2521	20.7<br>[18.7, 22.9]<br>p=< .001	-11.3<br>[-14.8, -7.8]<br>p=< .001
PHE>TYR	0.0014<br>< .001<br>< .001	8.6<br>[7.9, 9.3]	8.5<br>[7.9, 9.2]	7.8<br>[7.2, 8.5]<br>p=0.1811	6.4<br>[5.9, 7]<br>p=< .001	-1.4<br>[-2.3, -0.5]<br>p=0.003	11.8<br>[11.1, 12.7]<br>p=< .001	8.1<br>[7.5, 8.7]<br>p=0.7626	-3.8<br>[-4.8, -2.8]<br>p=< .001
Methionine<br>ReMethylation	0.351<br>0.0286<br>0.1209	5.8<br>[3.9, 7.7]	5.2<br>[3.3, 7.2]	5.6<br>[3.6, 7.6]<br>p=1	5.9<br>[4, 7.9]<br>p=0.9838	0.3<br>[-2.3, 3]<br>p=0.9615	9<br>[7.1, 10.9]<br>p=0.0176	6<br>[4.1, 7.8]<br>p=0.9817	-3<br>[-5.7, -0.4]<br>p=0.0524

Whole Body Production as mean: µmol/min [95% CI]. Statistics (JASP) by Generalized Linear Mixed Model (Family: Gaussian, Link: Identify or Log) with studygroup, studyday and pig weight as fixed effects variables and pig ID as random effects grouping factor. Post-hoc testing p values are corrected by Tukey. Group1 are the pigs that received EAA feeding and Group2 are the pigs that received TAA feeding post-sepsis. P ANOVA is the p-value of the main effect group.

Supplemental Table 4: Plasma Clearance of amino acids

Amino Acid	P ANOVA	Presepsis		Day 3			Day 7		
	Group<br>Time<br>Group*Time	Group1	Group2	EAA	TAA	TAA-EAA	EAA	TAA	TAA-EAA
Arginine	0.0027<br>< .001<br>< .001	2.03<br>[1.8, 2.28]	1.88<br>[1.66, 2.12]	1.91<br>[1.68, 2.16]<br>p=0.8643	1.53<br>[1.34, 1.75]<br>p=0.0182	-0.38<br>[-0.67, -0.09]<br>p=0.0209	2.78<br>[2.5, 3.09]<br>p=< .001	1.61<br>[1.42, 1.83]<br>p=0.163	-1.17<br>[-1.52, -0.82]<br>p=< .001
Citrulline	0.1765<br>< .001<br>0.1703	5.03<br>[4.36, 5.8]	4.1<br>[3.5, 4.81]	2.82<br>[2.34, 3.39]<br>p=< .001	2.42<br>[1.99, 2.95]<br>p=< .001	-0.39<br>[-1.08, 0.29]<br>p=0.4535	2.65<br>[2.19, 3.21]<br>p=< .001	2.08<br>[1.67, 2.6]<br>p=< .001	-0.57<br>[-1.26, 0.12]<br>p=0.1966
Glutamate	0.1765<br>< .001<br>0.1703	5.03<br>[4.36, 5.8]	4.1<br>[3.5, 4.81]	2.82<br>[2.34, 3.39]<br>p=< .001	2.42<br>[1.99, 2.95]<br>p=< .001	-0.39<br>[-1.08, 0.29]<br>p=0.4535	2.65<br>[2.19, 3.21]<br>p=< .001	2.08<br>[1.67, 2.6]<br>p=< .001	-0.57<br>[-1.26, 0.12]<br>p=0.1966
Glutamine	0.1428<br>< .001<br>< .001	0.43<br>[0.39, 0.48]	0.4<br>[0.36, 0.45]	0.26<br>[0.23, 0.3]<br>p=< .001	0.31<br>[0.27, 0.35]<br>p=< .001	0.05<br>[0, 0.09]<br>p=0.0606	0.3<br>[0.27, 0.34]<br>p=< .001	0.38<br>[0.34, 0.43]<br>p=0.8403	0.08<br>[0.03, 0.13]<br>p=0.0055
Glycine	1<br>< .001<br>1	0.7<br>[0.51, 0.96]	0.62<br>[0.43, 0.89]	0.11<br>[0.08, 0.16]<br>p=< .001	0.11<br>[0.07, 0.16]<br>p=< .001	-0.01<br>[-0.05, 0.03]<br>p=0.8999	0.11<br>[0.08, 0.15]<br>p=< .001	0.09<br>[0.06, 0.14]<br>p=< .001	-0.02<br>[-0.06, 0.02]<br>p=0.5545
Histidine	0.233<br>< .001<br>< .001	2.03<br>[1.71, 2.4]	2.06<br>[1.74, 2.45]	2.44<br>[2.06, 2.88]<br>p=0.0369	2.18<br>[1.85, 2.58]<br>p=0.9581	-0.25<br>[-0.78, 0.27]<br>p=0.5641	2.22<br>[1.89, 2.62]<br>p=0.7557	3.7<br>[3.2, 4.27]<br>p=< .001	1.48<br>[0.84, 2.11]<br>p=< .001
Hydroxy proline	1<br>< .001<br>1	0.26<br>[0.23, 0.3]	0.23<br>[0.2, 0.27]	0.19<br>[0.16, 0.23]<br>p=< .001	0.22<br>[0.19, 0.25]<br>p=0.9213	0.02<br>[-0.02, 0.07]<br>p=0.462	0.16<br>[0.13, 0.19]<br>p=< .001	0.2<br>[0.17, 0.23]<br>p=0.1806	0.04<br>[0, 0.08]<br>p=0.0989
Isoleucine	0.4994<br>< .001<br>0.5204	0.95<br>[0.85, 1.06]	0.89<br>[0.8, 1]	0.65<br>[0.57, 0.74]<br>p=< .001	0.59<br>[0.52, 0.67]<br>p=< .001	-0.06<br>[-0.16, 0.05]<br>p=0.5057	0.73<br>[0.65, 0.82]<br>p=< .001	0.67<br>[0.59, 0.75]<br>p=< .001	-0.06<br>[-0.17, 0.05]<br>p=0.471
Leucine	0.8888<br>< .001<br>0.8401	0.96<br>[0.85, 1.08]	0.99<br>[0.88, 1.11]	1.25<br>[1.11, 1.4]<br>p=< .001	1.29<br>[1.16, 1.43]<br>p=< .001	0.04<br>[-0.15, 0.23]<br>p=0.9167	0.94<br>[0.84, 1.06]<br>p=0.9998	0.93<br>[0.83, 1.04]<br>p=0.7814	-0.01<br>[-0.16, 0.13]<br>p=0.977
Lysine	0.0685<br>< .001<br>< .001	5.97<br>[4.81, 7.41]	6.57<br>[5.35, 8.06]	1.07<br>[0.78, 1.47]<br>p=< .001	2.02<br>[1.6, 2.55]<br>p=< .001	0.95<br>[0.39, 1.51]<br>p=0.0018	0.91<br>[0.64, 1.3]<br>p=< .001	1.51<br>[1.18, 1.95]<br>p=< .001	0.6<br>[0.1, 1.1]<br>p=0.0349

Methionine1	< .001<br>< .001<br>< .001	1.01<br>[0.87, 1.18]	1.04<br>[0.88, 1.22]	0.64<br>[0.54, 0.77]<br>p=< .001	1<br>[0.85, 1.17]<br>p=0.9745	0.35<br>[0.18, 0.52]<br>p=< .001	0.46<br>[0.38, 0.57]<br>p=< .001	0.94<br>[0.79, 1.1]<br>p=0.386	0.47<br>[0.3, 0.64]<br>p=< .001
Methionine3	0.0029<br>< .001<br>< .001	1.2<br>[1.04, 1.4]	1.21<br>[1.03, 1.42]	0.82<br>[0.69, 0.97]<br>p=< .001	1.26<br>[1.07, 1.47]<br>p=0.9748	0.44<br>[0.22, 0.66]<br>p=< .001	0.61<br>[0.51, 0.74]<br>p=< .001	1.13<br>[0.96, 1.32]<br>p=0.753	0.52<br>[0.32, 0.72]<br>p=< .001
Ornithine	0.806<br>< .001<br>0.002	0.36<br>[0.33, 0.39]	0.33<br>[0.3, 0.36]	0.27<br>[0.25, 0.3]<br>p=< .001	0.29<br>[0.27, 0.32]<br>p=0.001	0.02<br>[-0.01, 0.05]<br>p=0.391	0.23<br>[0.21, 0.25]<br>p=< .001	0.23<br>[0.21, 0.25]<br>p=< .001	0<br>[-0.03, 0.03]<br>p=0.997
Phenylalanine	0.28<br>< .001<br>0.868	1.09<br>[0.98, 1.22]	1.16<br>[1.05, 1.29]	1.03<br>[0.92, 1.15]<br>p=0.626	1.11<br>[1, 1.23]<br>p=0.731	0.08<br>[-0.08, 0.24]<br>p=0.57	0.85<br>[0.76, 0.95]<br>p=< .001	0.94<br>[0.84, 1.04]<br>p=< .001	0.09<br>[-0.05, 0.23]<br>p=0.378
Taurine	0.243<br>< .001<br>< .001	0.26<br>[0.23, 0.3]	0.25<br>[0.22, 0.29]	0.25<br>[0.22, 0.29]<br>p=0.986	0.26<br>[0.23, 0.3]<br>p=0.988	0.01<br>[-0.04, 0.05]<br>p=0.924	0.29<br>[0.26, 0.33]<br>p=0.214	0.4<br>[0.35, 0.45]<br>p=< .001	0.11<br>[0.05, 0.16]<br>p=< .001
Tau-methyl-histidine	0.221<br>< .001<br>0.011	0.06<br>[0.06, 0.07]	0.06<br>[0.06, 0.07]	0.06<br>[0.06, 0.07]<br>p=1	0.07<br>[0.06, 0.07]<br>p=0.24	0<br>[0, 0.01]<br>p=0.756	0.06<br>[0.05, 0.06]<br>p=< .001	0.06<br>[0.06, 0.07]<br>p=1	0.01<br>[0, 0.01]<br>p=0.207
Threonine	< .001<br>< .001<br>< .001	0.93<br>[0.77, 1.11]	1.07<br>[0.89, 1.29]	0.2<br>[0.15, 0.27]<br>p=< .001	0.59<br>[0.49, 0.72]<br>p=< .001	0.39<br>[0.26, 0.52]<br>p=< .001	0.15<br>[0.11, 0.22]<br>p=< .001	0.39<br>[0.31, 0.49]<br>p=< .001	0.24<br>[0.13, 0.34]<br>p=< .001
Tryptophan	0.164<br>< .001<br>< .001	0.81<br>[0.67, 0.97]	0.77<br>[0.63, 0.93]	0.84<br>[0.7, 1.02]<br>p=0.972	0.85<br>[0.71, 1.02]<br>p=0.522	0<br>[-0.21, 0.21]<br>p=0.999	0.73<br>[0.61, 0.88]<br>p=0.76	1.36<br>[1.15, 1.61]<br>p=< .001	0.63<br>[0.37, 0.88]<br>p=< .001
Tyrosine	< .001<br>< .001<br>< .001	1.86<br>[1.7, 2.04]	1.81<br>[1.64, 1.99]	1.1<br>[0.97, 1.25]<br>p=< .001	1.88<br>[1.71, 2.06]<br>p=0.93	0.78<br>[0.56, 1]<br>p=< .001	1.04<br>[0.92, 1.18]<br>p=< .001	1.51<br>[1.37, 1.67]<br>p=0.002	0.47<br>[0.28, 0.67]<br>p=< .001
Valine	0.326<br>0.036<br>0.431	0.54<br>[0.47, 0.62]	0.56<br>[0.49, 0.63]	0.53<br>[0.47, 0.61]<br>p=1	0.63<br>[0.56, 0.71]<br>p=0.251	0.09<br>[-0.01, 0.2]<br>p=0.148	0.5<br>[0.44, 0.57]<br>p=0.809	0.52<br>[0.46, 0.59]<br>p=0.9	0.02<br>[-0.07, 0.12]<br>p=0.851

Plasma Clearances are mean in Liter/min [95% CI]. Statistics (JASP) by Generalized Linear Mixed Model (Family: Gaussian, Link: Identify or Log) with studygroup, studyday and pig weight as fixed effects variables and pig ID as random effects grouping factor. Post hoc testing p values are corrected by Tukey. Group1 are the pigs that after sepsis will receive the EAA nutrition and Group2 are the pigs that after sepsis will receive the TAA nutrition. P ANOVA is the p-value of the main effect group.

Supplemental Table 5: Size of pool 1 (Q1: Represents extracellular pool) of amino acids

Amino Acid	P ANOVA	Presepsis		Day 3			Day 7		
	Group<br>Time<br>Group*Time	Group1	Group2	EAA	TAA	TAA-EAA	EAA	TAA	TAA-EAA
Arginine	1<br>< .001<br>1	1604<br>[1475, 1745]	1673<br>[1549, 1807]	1080<br>[966, 1207]<br>p=< .001	1143<br>[1038, 1259]<br>p=< .001	63<br>[-99, 225]<br>p=0.6925	1290<br>[1169, 1423]<br>p=< .001	1399<br>[1278, 1532]<br>p=< .001	110<br>[-80, 299]<br>p=0.4476
Citrulline	1<br>1<br>1	549<br>[496, 607]	527<br>[435, 640]	571<br>[514, 634]<br>p=0.9804	498<br>[453, 547]<br>p=0.9929	-73<br>[-138, -9]<br>p=0.0523	760<br>[693, 833]<br>p=< .001	639<br>[535, 763]<br>p=0.0076	-121<br>[-218, -23]<br>p=0.03
Glutamate	1<br>< .001<br>1	2769<br>[2131, 3596]	4745<br>[3921, 5743]	4396<br>[3550, 5445]<br>p=< .001	4802<br>[4081, 5651]<br>p=1	406<br>[-933, 1745]<br>p=0.7999	6284<br>[5459, 7233]<br>p=< .001	5531<br>[4781, 6398]<br>p=0.336	-753<br>[-2070, 565]<br>p=0.4565
Glutamine	< .001<br>< .001<br>< .001	8056<br>[7530, 8620]	7954<br>[7440, 8502]	6674<br>[6190, 7195]<br>p=< .001	5682<br>[5256, 6144]<br>p=< .001	-991<br>[-1650, -332]<br>p=0.0064	7433<br>[6923, 7981]<br>p=0.099	6027<br>[5595, 6491]<br>p=< .001	-1406<br>[-2103, -710]<br>p=< .001
Glycine	0.0386<br>< .001<br>< .001	9284<br>[8716, 9889]	9045<br>[8478, 9650]	7001<br>[6491, 7550]<br>p=< .001	9637<br>[9036, 10278]<br>p=0.2711	2637<br>[1798, 3475]<br>p=< .001	8119<br>[7583, 8694]<br>p=< .001	11049<br>[10420, 11716]<br>p=< .001	2930<br>[2042, 3818]<br>p=< .001
Histidine	< .001<br>< .001<br>< .001	1226<br>[1137, 1322]	1164<br>[1082, 1251]	1341<br>[1245, 1445]<br>p=0.1731	927<br>[847, 1015]<br>p=< .001	-414<br>[-541, -286]<br>p=< .001	1853<br>[1727, 1988]<br>p=< .001	1201<br>[1113, 1296]<br>p=0.9836	-652<br>[-810, -494]<br>p=< .001
Hydroxy proline	0.0276<br>< .001<br>< .001	713<br>[628, 810]	663<br>[579, 758]	442<br>[378, 516]<br>p=< .001	487<br>[420, 566]<br>p=< .001	45<br>[-56, 147]<br>p=0.6191	316<br>[261, 383]<br>p=< .001	475<br>[408, 552]<br>p=< .001	159<br>[64, 254]<br>p=0.0021
Isoleucine	1<br>< .001<br>1	1356<br>[1230, 1495]	1397<br>[1266, 1541]	1245<br>[1123, 1382]<br>p=0.2578	1498<br>[1365, 1645]<br>p=0.4173	253<br>[138, 369]<br>p=< .001	1434<br>[1297, 1585]<br>p=0.7782	1607<br>[1457, 1772]<br>p=0.0071	173<br>[22, 324]<br>p=0.048
Leucine	1<br>< .001<br>1	2654<br>[2515, 2801]	2569<br>[2417, 2729]	1946<br>[1808, 2094]<br>p=< .001	1991<br>[1861, 2131]<br>p=< .001	45<br>[-144, 234]<br>p=0.8689	2768<br>[2625, 2919]<br>p=0.8206	2636<br>[2497, 2783]<br>p=0.981	-132<br>[-336, 72]<br>p=0.3673
Lysine	< .001<br>< .001<br>< .001	3380<br>[2964, 3854]	3147<br>[2788, 3552]	6934<br>[6348, 7573]<br>p=< .001	3620<br>[3225, 4063]<br>p=0.3963	-3314<br>[-4053, -2575]<br>p=< .001	10067<br>[9360, 10828]<br>p=< .001	5789<br>[5343, 6272]<br>p=< .001	-4279<br>[-5134, -3423]<br>p=< .001

Methionine1	< .001<br>< .001<br>< .001	741<br>[665, 825]	688<br>[618, 764]	638<br>[565, 720]<br>p=0.0372	445<br>[388, 509]<br>p=< .001	-193<br>[-291, -95]<br>p=< .001	1022<br>[926, 1127]<br>p=< .001	613<br>[550, 683]<br>p=0.2805	-408<br>[-528, -288]<br>p=< .001
Methionine3	1<br>< .001<br>< .001	755<br>[671, 849]	636<br>[562, 720]	645<br>[567, 734]<br>p=0.0195	437<br>[378, 506]<br>p=< .001	-208<br>[-307, -108]<br>p=< .001	990<br>[885, 1107]<br>p=< .001	598<br>[530, 674]<br>p=0.8929	-392<br>[-523, -261]<br>p=< .001
Ornithine	0.2332<br>< .001<br>0.0256	446<br>[412, 479]	450<br>[416, 485]	322<br>[287, 358]<br>p=< .001	298<br>[263, 333]<br>p=< .001	-24<br>[-73, 24]<br>p=0.5431	413<br>[378, 449]<br>p=0.706	489<br>[455, 523]<br>p=0.4834	75<br>[26, 125]<br>p=0.0057
Phenylalanine	1<br>< .001<br>0.0265	1132<br>[1045, 1226]	1025<br>[946, 1111]	892<br>[806, 987]<br>p=< .001	815<br>[736, 903]<br>p=< .001	-77<br>[-200, 46]<br>p=0.3915	1448<br>[1352, 1551]<br>p=< .001	1161<br>[1074, 1254]<br>p=0.1051	-287<br>[-421, -154]<br>p=< .001
Taurine	< .001<br>< .001<br>< .001	829<br>[758, 900]	784<br>[713, 856]	674<br>[601, 746]<br>p=0.0022	528<br>[456, 600]<br>p=< .001	-146<br>[-235, -57]<br>p=0.0027	737<br>[663, 811]<br>p=0.2728	446<br>[375, 518]<br>p=< .001	-291<br>[-390, -192]<br>p=< .001
Tau-methyl-<br>histidine	< .001<br>< .001<br>< .001	829<br>[758, 900]	784<br>[713, 856]	674<br>[601, 746]<br>p=< .001	528<br>[456, 600]<br>p=0.6698	-146<br>[-235, -57]<br>p=0.0027	737<br>[663, 811]<br>p=< .001	446<br>[375, 518]<br>p=< .001	-291<br>[-390, -192]<br>p=< .001
Threonine	< .001<br>< .001<br>< .001	2011<br>[1593, 2429]	1804<br>[1380, 2227]	8632<br>[7930, 9333]<br>p=< .001	2249<br>[1831, 2666]<br>p=0.4752	-6383<br>[-7177, -5589]<br>p=< .001	8859<br>[6969, 10748]<br>p=< .001	3806<br>[3385, 4226]<br>p=< .001	-5053<br>[-6989, -3118]<br>p=< .001
Tryptophan	1<br>< .001<br>< .001	427<br>[399, 458]	368<br>[343, 395]	343<br>[314, 374]<br>p=< .001	319<br>[293, 347]<br>p=0.0423	-24<br>[-64, 16]<br>p=0.4166	462<br>[431, 495]<br>p=0.4971	327<br>[300, 356]<br>p=0.1575	-135<br>[-178, -93]<br>p=< .001
Tyrosine	< .001<br>< .001<br>< .001	1070<br>[991, 1155]	1073<br>[996, 1156]	1286<br>[1195, 1383]<br>p=< .001	842<br>[772, 918]<br>p=< .001	-444<br>[-561, -327]<br>p=< .001	1551<br>[1455, 1653]<br>p=< .001	1174<br>[1096, 1257]<br>p=0.1819	-377<br>[-505, -249]<br>p=< .001
Valine	< .001<br>< .001<br>< .001	2681<br>[2490, 2886]	2512<br>[2333, 2706]	2508<br>[2313, 2719]<br>p=0.5242	1915<br>[1748, 2099]<br>p=< .001	-593<br>[-854, -331]<br>p=< .001	3338<br>[3110, 3583]<br>p=< .001	2600<br>[2420, 2794]<br>p=0.9689	-738<br>[-1040, -437]<br>p=< .001

Pool size as mean: µmol [95% CI]. Statistics (JASP) by Generalized Linear Mixed Model (Family: Gaussian, Link: Identify or Log) with studygroup, studyday and pig weight as fixed effects variables and pig ID as random effects grouping factor. Post-hoc testing p values are corrected by Tukey. Group1 are the pigs that received EAA feeding and Group2 are the pigs that received TAA feeding post-sepsis. P ANOVA is the p-value of the main effect group.

Supplemental Table 6: Size of pool 2 (Q2: Represents intracellular pool) of amino acids

Amino Acid	P ANOVA	Presepsis		Day 3			Day 7		
	Group<br>Time<br>Group*Time	Group1	Group2	EAA	TAA	TAA-EAA	EAA	TAA	TAA-EAA
Arginine	0.0012<br>< .001<br>< .001	19906<br>[16047, 23765]	18615<br>[14892, 22338]	22509<br>[18660, 26358]<br>p=0.7972	12321<br>[8698, 15945]<br>p=0.0163	-10188<br>[-15269, -5106]<br>p=< .001	36168<br>[30907, 41429]<br>p=< .001	20991<br>[17419, 24564]<br>p=0.8511	-15176<br>[-21533, -8820]<br>p=< .001
Citrulline	0.8797<br>< .001<br>0.0048	1972<br>[1833, 2122]	1954<br>[1811, 2108]	1651<br>[1519, 1795]<br>p=< .001	1571<br>[1443, 1709]<br>p=< .001	-81<br>[-271, 110]<br>p=0.6497	1977<br>[1835, 2131]<br>p=1	1703<br>[1577, 1839]<br>p=0.0085	-274<br>[-475, -74]<br>p=0.0147
Glutamine	0.6116<br>< .001<br>0.2721	45497<br>[41559, 49436]	42522<br>[38534, 46510]	24144<br>[20106, 28183]<br>p=< .001	25526<br>[21563, 29489]<br>p=< .001	1381<br>[-3640, 6403]<br>p=0.8317	31120<br>[26956, 35283]<br>p=< .001	31469<br>[27488, 35449]<br>p=< .001	349<br>[-5253, 5952]<br>p=0.9905
Glutamate	0.9548<br>< .001<br>0.6169	6308<br>[-15042, 27659]	946<br>[-26345, 28237]	89905<br>[73014, 106796]<br>p=< .001	86396<br>[70384, 102409]<br>p=< .001	-3508<br>[-25916, 18899]<br>p=0.9419	112391<br>[95880, 128902]<br>p=< .001	110340<br>[94801, 125880]<br>p=< .001	-2051<br>[-24716, 20615]<br>p=0.9802
Glycine	0.7134<br>< .001<br>0.663	381907<br>[255657, 508156]	426959<br>[295995, 557924]	13119<br>[-109867, 136105]<br>p=< .001	18243<br>[-99968, 136454]<br>p=< .001	5124<br>[-160244, 170493]<br>p=0.9977	22510<br>[-103683, 148703]<br>p=< .001	25171<br>[-92357, 142700]<br>p=< .001	2661<br>[-169827, 175149]<br>p=0.9994
Histidine	0.0807<br>< .001<br>0.9941	6382<br>[5895, 6910]	6431<br>[5960, 6940]	4575<br>[4086, 5122]<br>p=< .001	5020<br>[4579, 5504]<br>p=< .001	446<br>[-182, 1074]<br>p=0.3017	5433<br>[4953, 5960]<br>p=0.0338	5148<br>[4701, 5637]<br>p=< .001	-285<br>[-889, 318]<br>p=0.5827
Hydroxy proline	1<br>< .001<br>1	2801<br>[2232, 3514]	3090<br>[2376, 4018]	1387<br>[1010, 1903]<br>p=< .001	2867<br>[2202, 3734]<br>p=0.9884	1480<br>[742, 2219]<br>p=< .001	536<br>[309, 933]<br>p=< .001	1849<br>[1324, 2581]<br>p=0.008	1312<br>[639, 1986]<br>p=< .001
Isoleucine	1<br>< .001<br>1	12558<br>[11325, 13925]	10432<br>[9392, 11586]	7103<br>[6124, 8240]<br>p=< .001	6012<br>[5160, 7005]<br>p=< .001	-1091<br>[-2504, 322]<br>p=0.2432	9171<br>[8124, 10352]<br>p=< .001	7547<br>[6655, 8558]<br>p=< .001	-1624<br>[-3067, -181]<br>p=0.054
Leucine	< .001<br>< .001<br>0.0146	23061<br>[21177, 25112]	21745<br>[19891, 23772]	18335<br>[16562, 20298]<br>p=< .001	18267<br>[16562, 20148]<br>p=0.006	-68<br>[-2507, 2371]<br>p=0.9981	21704<br>[19800, 23790]<br>p=0.7824	20518<br>[18779, 22418]<br>p=0.8296	-1186<br>[-3857, 1485]<br>p=0.6209
Lysine	< .001<br>< .001<br>0.0841	52479<br>[46395, 59361]	51336<br>[45094, 58443]	27914<br>[24336, 32019]<br>p=< .001	22894<br>[20024, 26174]<br>p=< .001	-5021<br>[-9508, -533]<br>p=0.0559	37848<br>[33431, 42850]<br>p=< .001	27668<br>[24410, 31361]<br>p=< .001	-10180<br>[-15581, -4780]<br>p=< .001

Methionine1	< .001<br>< .001<br>< .001	5104<br>[4576, 5692]	4611<br>[4106, 5178]	2315<br>[1924, 2786]<br>p=< .001	3036<br>[2615, 3525]<br>p=< .001	721<br>[102, 1341]<br>p=0.0445	2811<br>[2379, 3321]<br>p=< .001	3810<br>[3346, 4338]<br>p=0.0341	999<br>[324, 1674]<br>p=0.0075
Methionine3	0.1986<br>< .001<br>< .001	4880<br>[4385, 5432]	4386<br>[3933, 4892]	2765<br>[2404, 3181]<br>p=< .001	3642<br>[3237, 4098]<br>p=0.0031	876<br>[300, 1453]<br>p=0.0058	3489<br>[3078, 3954]<br>p=< .001	4192<br>[3763, 4671]<br>p=0.938	704<br>[83, 1324]<br>p=0.052
Ornithine	0.7049<br>< .001<br>0.1983	2138<br>[2047, 2234]	2190<br>[2088, 2298]	1598<br>[1514, 1686]<br>p=< .001	1492<br>[1413, 1574]<br>p=< .001	-106<br>[-218, 6]<br>p=0.123	1725<br>[1630, 1826]<br>p=< .001	1874<br>[1779, 1974]<br>p=< .001	149<br>[15, 283]<br>p=0.0568
Phenylalanine	< .001<br>< .001<br>< .001	8166<br>[7472, 8925]	7916<br>[7258, 8634]	5371<br>[4839, 5962]<br>p=< .001	5776<br>[5254, 6350]<br>p=< .001	405<br>[-374, 1184]<br>p=0.5216	6084<br>[5528, 6695]<br>p=< .001	5939<br>[5423, 6504]<br>p=< .001	-145<br>[-935, 645]<br>p=0.9214
Taurine	< .001<br>< .001<br>0.0062	3224<br>[3019, 3442]	3033<br>[2832, 3249]	2305<br>[2130, 2493]<br>p=< .001	1837<br>[1681, 2006]<br>p=< .001	-468<br>[-669, -267]<br>p=< .001	3015<br>[2806, 3240]<br>p=0.2927	2422<br>[2243, 2616]<br>p=< .001	-593<br>[-839, -347]<br>p=< .001
Tau-methyl-histidine	0.0381<br>< .001<br>0.0047	57.2<br>[49.8, 65.7]	54.5<br>[47.5, 62.7]	90<br>[79.4, 101.9]<br>p=< .001	64.9<br>[57, 73.9]<br>p=0.0403	-25.1<br>[-38, -12.2]<br>p=< .001	87.3<br>[77.2, 98.7]<br>p=< .001	69.1<br>[61, 78.3]<br>p=0.0016	-18.2<br>[-31.7, -4.7]<br>p=0.0166
Threonine	< .001<br>< .001<br>< .001	17309<br>[15542, 19076]	16754<br>[14964, 18545]	11941<br>[10095, 13787]<br>p=< .001	9279<br>[7511, 11047]<br>p=< .001	-2663<br>[-5127, -198]<br>p=0.0673	27125<br>[25156, 29094]<br>p=< .001	9919<br>[8174, 11664]<br>p=< .001	-17206<br>[-19836, -14575]<br>p=< .001
Tryptophan	< .001<br>1<br>1	2289<br>[2025, 2587]	2255<br>[1979, 2569]	1688<br>[1470, 1938]<br>p=< .001	1778<br>[1546, 2044]<br>p=< .001	90<br>[-167, 346]<br>p=0.7436	1954<br>[1703, 2241]<br>p=0.0554	2575<br>[2256, 2939]<br>p=0.0579	621<br>[255, 988]<br>p=0.0018
Tyrosine	1<br>1<br>1	9491<br>[8075, 11156]	8766<br>[7433, 10338]	5387<br>[4503, 6444]<br>p=< .001	6469<br>[5448, 7680]<br>p=< .001	1082<br>[372, 1792]<br>p=0.0057	6956<br>[5825, 8307]<br>p=< .001	7448<br>[6272, 8845]<br>p=0.0022	492<br>[-437, 1420]<br>p=0.5091
Valine	0.3703<br>< .001<br>0.1241	9263<br>[8712, 9850]	8840<br>[8311, 9402]	7412<br>[6897, 7965]<br>p=< .001	7533<br>[7044, 8057]<br>p=< .001	121<br>[-606, 849]<br>p=0.9345	9795<br>[9225, 10400]<br>p=0.4484	8902<br>[8392, 9442]<br>p=1	-893<br>[-1678, -108]<br>p=0.0508

Pool size as mean: µmol [95% CI]. Statistics (JASP) by Generalized Linear Mixed Model (Family: Gaussian, Link: Identify or Log) with studygroup, studyday and pig weight as fixed effects variables and pig ID as random effects grouping factor. Post hoc testing p values are corrected by Tukey. Bold is p<0.05. GGroup1 are the pigs that received EAA feeding and Group2 are the pigs that received TAA feeding post-sepsis. P ANOVA is the p-value of the main effect group.
